# Genetic markers for non-syndromic orofacial clefts in populations of European ancestry: a meta-analysis

**DOI:** 10.1038/s41598-021-02159-5

**Published:** 2022-01-24

**Authors:** Lara Slavec, Nataša Karas Kuželički, Igor Locatelli, Ksenija Geršak

**Affiliations:** 1 University Medical Centre Ljubljana, Division of Gynaecology and Obstetrics, Research Unit, Ljubljana, Slovenia; 2 University of Ljubljana, Faculty of Pharmacy, Department of Clinical Biochemistry, Ljubljana, Slovenia; 3 University of Ljubljana, Faculty of Pharmacy, Department of Social Pharmacy, Ljubljana, Slovenia; 4 University of Ljubljana, Faculty of Medicine, Department of Gynaecology and Obstetrics, Ljubljana, Slovenia

**Keywords:** Risk factors, Epidemiology, Genetics

## Abstract

To date, the involvement of various genetic markers in the aetiopathogenesis of non-syndromic orofacial cleft (nsOFC) has been extensively studied. In the present study, we focused on studies performed on populations of European ancestry to systematically review the available literature to define relevant genetic risk factors for nsOFC. Eligible studies were obtained by searching Ovid Medline and Ovid Embase. We gathered the genetic markers from population-based case–control studies on nsOFC, and conducted meta-analysis on the repeatedly reported markers. Whenever possible, we performed stratified analysis based on different nsOFC phenotypes, using allelic, dominant, recessive and overdominant genetic models. Effect sizes were expressed as pooled odds ratios (ORs) with 95% confidence intervals (CIs), and p ≤ 0.05 were considered statistically significant. A total of 84 studies were eligible for this systematic review, with > 700 markers included. Of these, 43 studies were included in the meta-analysis. We analysed 47 genetic variants in 30 genes/loci, which resulted in 226 forest plots. There were statistically significant associations between at least one of the nsOFC phenotypes and 19 genetic variants in 13 genes/loci. These data suggest that *IRF6*, *GRHL3*, 8q24, *VAX1*, *TGFA*, *FOXE1*, *ABCA4*, *NOG*, *GREM1*, *AXIN2*, *DVL2*, *WNT3A* and *WNT5A* have high potential as biomarkers of nsOFC in populations of European descent. Although other meta-analyses that included European samples have been performed on a limited number of genetic variants, this study represents the first meta-analysis of all genetic markers that have been studied in connection with nsOFC in populations of European ancestry.

## Introduction

Non-syndromic orofacial cleft (nsOFC) is a birth defect that is characterised by incomplete division of the oral and nasal cavities, without any other anomalies present. OFC is considered the most common form of congenital craniofacial malformation. The worldwide incidence ranges from around 1/2500 to 1/500 births, making OFC one of the most frequent congenital developmental defects^1–3^. The rate of occurrence varies substantially based on geographic origin, ethnicity and race. In general, with some exceptions, populations of Asian and Native American descent have the highest risk of developing OFC, while populations of European ancestry have intermediate risk, and African-derived populations have the lowest risk. In various European countries where White people predominate, OFC occurs at an incidence rate from 1/1000 to 1/500 live births^1^.

Non-syndromic OFC develops during embryogenesis as a result of incomplete growth and fusion of the soft and/or hard facial tissues, and the subsequent abnormal formation of some parts of the face and oral cavity (i.e., nose, lip, alveolus, palate)^4^. NsOFC is generally divided into groups based on the structures affected, as: cleft lip only; cleft lip and alveolus; cleft lip, alveolus and palate; and cleft palate only (nsCPO). Due to the similar epidemiological features and embryological mechanisms involved, non-syndromic cleft lip only, non-syndromic cleft lip and alveolus, and non-syndromic cleft lip, alveolus and palate are most often investigated together as one group, which is indicated as cleft lip with or without cleft palate (nsCL/P), while nsCPO is believed to have a different, but overlapping, aetiology^5^.

It is generally accepted that nsOFC is a complex multifactorial disorder that arises from interactions between multiple genetic and environmental factors. Various studies have reported high incidence of nsOFC within individual families and in the offspring of patients with nsOFC^6^. Furthermore, a higher level of concordance has been observed for monozygotic twins (40–60%) over dizygotic twins (3–5%), which suggests that the genetic component of nsOFC is strong^7,8^. Differences in the prevalence of nsOFC between ethnic groups and populations further confirm the involvement of genetics in this incidence.

Although the full pattern of inheritance is still unknown, nsOFC has been linked to numerous genes, including some that encode transcription factors and growth factors, and some that are involved in cellular signalling and metabolism of nutrients and xenobiotics^9^. To date, many genetic association studies have been performed to investigate the involvement of such candidate genes in nsOFC^10–16^. While several of these studies have shown consistent and replicable genetic association between some genes and nsOFC^10–12^, many of them have failed to demonstrate that^13–16^. One reason might be that studies have been carried out on different populations or racial groups, and using insufficient sample sizes.

Several systematic reviews and meta-analyses have been performed for selected polymorphisms for some of the genes implicated (e.g., *IRF6*, *MSX1*, *MTHFR*)^17–19^, which have used pooled data from different ethnic groups. One of these meta-analyses was aimed at identification of all of the genetic markers associated with nsOFC in a Brazilian population^20^, which exhibited high levels of racial and ethnic admixture. However, in the context of the genetics of nsOFC, the data of Brazilian study are not useful when studying populations of European ancestry. Thus, obtaining information about all the genes/genetic variants associated with nsOFC in populations of European ancestry is of importance.

In individuals with nsOFC, surgical, dental, feeding, breathing, speech, hearing and/or psychological interventions represent a substantial burden to health systems^21^. The identification of nsOFC genetic markers should lead to better understanding of craniofacial development and to improved prenatal diagnostics and genetic counselling, thus enhancing nsOFC preventive programmes. Hence, we conducted this study to systematically review the available literature for relevant population-based case–control studies, and to gather together all of the genetic markers that have been examined in connection with nsOFC in populations of European descent.

Traditionally, the term ‘Caucasian’ was used as a racial classification for White people that came from different ethnic groups. Zhang et al. addressed the inconsistency in the race and ethnic classification in pharmacogenetic studies^22^. When they reviewed the literature and evaluated the application of current racial and ethnic categories, they found ‘Caucasian’ (‘White’) category highly heterogeneous^22^. The term ‘Caucasian’ is now obsolete. However, in the reviewed genetic studies, researchers classified the studied population as ‘Caucasian’ or ‘White’ when the population was European, Australian, Middle Eastern, or North/South African^22^. To reduce the genetic heterogeneity, this study was limited to populations of European ancestry only. The meta-analysis was carried out on repeatedly reported markers, to identify potential genetic risk factors for nsOFC. Although other meta-analyses that included samples of White people have been performed on a limited number of genetic variants^18,19,23–26^, to the best of our knowledge, this study represents the first systematic review and meta-analysis of all genetic markers that have been examined in connection with nsOFC in populations of European descent only.

## Materials and methods

The present systematic review and meta-analysis was performed according to the Preferred Reporting Items for Systematic Reviews and Meta-Analyses (PRISMA) checklist^27^. The protocol was registered with PROSPERO (international prospective register of systematic reviews) under the accession number CRD42020163436.

### Search strategy

A literature search was conducted using two electronic bibliographic databases: Ovid Medline and Ovid Embase. The search was limited to human studies, and covered publications up to 20 June, 2019. There were no restrictions regarding language or publication period. Briefly, the search included the following terms: (cleft lip OR cleft palate OR oral cleft OR orofacial cleft OR cheiloschisis OR gnathoschisis OR palatoschisis OR cheilognathopalatoschisis) and (nonsyndromic OR non-syndromic OR isolated) and (polymorphism OR gene OR genotype OR nucleotide OR SNP OR variant OR SNV OR marker OR factor OR mutation OR allele). The full literature search strategy is available from the registered protocol in the PROSPERO database. References included in previous meta-analyses that examined associations between genetic markers and nsOFC were also screened to identify any publications that might have been missed.

### Identification of eligible studies

#### Inclusion criteria

This review covered studies that investigated the association of genetic factors and nsOFC in populations of European ancestry. We used the ‘population, intervention, comparison, outcomes and study’ (PICOS) principle to define the research question and to set the inclusion criteria. Studies that met the following criteria were included in the systematic review:

##### Population

Patients with nsOFC that had European descent. In this review, the patients are considered as Europeans if they were residents of a European country, or of the United States, Canada, South America or Australia, and if their ancestors came from Europe and were White.

##### Intervention

Investigation of genetic factors (defined by ‘rs’ number or other identifiable name).

##### Comparison

The control group that comprised unrelated and unaffected individuals without any family history of OFC.

##### Outcome

Association of the selected genetic factors with the occurrence of nsOFC. The outcomes had to be reported as odds ratios (ORs) with 95% confidence intervals (CIs). Otherwise, there needed to be enough information for calculations of ORs with 95% CIs.

##### Study design

Population-based case–control studies on humans (observational genetic association studies); studies focussing on candidate genes.

#### Exclusion criteria

One of the main exclusion criteria was the unsuitable origin of the population included. Studies that did not include people of European ancestry, but included other populations that were considered White (e.g., Turkish, Iranian populations), were excluded. For studies with a mixed population from non-European countries, only those with > 90% of patients and controls of European descent were included. A lack of this information also resulted in study exclusion. Studies such as case reports, family-based studies, case series, reviews and meta-analyses, were not included. The following were also excluded: genome-wide association studies (GWAS); studies not concerning genetic markers (SNVs or indels); studies not focused on humans (e.g., in vitro or animal model studies); studies without a control group; studies that only investigated maternal genetic markers; studies that examined the genetics of syndromic OFC (or other diseases); and studies without adequate information (e.g., conference abstracts, short letters). Even though GWAS were not included, when they had a population-based replication phase on candidate genes/genetic variants, we did include the replication case–control sample in our study. Studies that looked only for DNA mutations in nsOFC patients that were not seen for a control group were also eliminated. Similarly for studies that examined rare polymorphisms without identifiable names, or for which the location on the chromosome was not determined. If two or more studies evaluated overlapping study populations, all of them were included in the systematic review, but only the study with the most information and the largest sample size was selected for inclusion in the meta-analysis.

### Study selection process and data extraction

After the literature search, the eligible studies were selected in a two-phase process. Initially, all of the titles and abstracts were screened. Articles recognised as not relevant were excluded. Secondly, the full-text versions of all remaining articles were read, for selection of those that matched the inclusion criteria. The following information was extracted from all of the eligible articles: first author; year of publication; population studied; name of the gene/loci or genetic marker inspected; genotyping method; numbers of cases and controls included in the study; and results of the study. If an article included different types of studies, only the information (e.g. number of cases and controls) regarding population-based case–control studies that focussed on candidate genes was extracted. If the studies examined different populations, only the information about populations of European ancestry was extracted. For all of the genetic markers investigated, the genes in which they are located (or are in their proximity) were also defined. When the marker accession number (i.e., ‘rs’ number) was not given, other published articles were examined for help using the PubMed database; and when the gene name was not given, dbSNP database^28^ was searched. Markers without identifiable names that could not be found in any other study were excluded. The human gene database GeneCards^29^ (based on human reference genome version GRCh38/hg38) was used to extract the name, chromosome number and exact genomic location for each gene included. All of these steps were carried out by one of the authors (L.S.) and were then cross-checked and re-evaluated by a second author (N.K.K.). When it was necessary to resolve disagreements, a third author was available (I.L.).

### Quality assessment of studies included

The risk of bias for all of the studies included in the systematic review was assessed using the Newcastle–Ottawa Scale (NOS). This is a frequently used method for estimation of the methodological quality of non-randomised studies (i.e., observational case–control studies). The NOS uses a ‘star system’ to rate the quality of individual studies based on three separate aspects: selection of the groups studied; their comparability; and assessment of the outcome^30^. Quality scores range from 0 stars (lowest quality) to 9 stars (highest quality). For the studies included, the NOS quality scores were defined independently by two authors (L.S., I.L.). A score ≥ 6 indicated good or high methodological quality, and therefore low risk of bias. Disagreements on the NOS quality scores were discussed and resolved with a third author (N.K.K.).

### Data synthesis and statistical analysis

All of the eligible studies were included in the systematic review. The genetic variants that were examined in these studies in connection with nsOFC were manually extracted. Furthermore, where two or more studies investigated the relationships between one genetic marker and nsOFC, meta-analysis was also conducted. The allelic frequencies or ORs with 95% CIs were obtained from these studies.

Genotyping counts were also collected, if available. The controls were checked for Hardy–Weinberg equilibrium (HWE). If information about the HWE was missing, this was calculated using Chi-squared tests, where p > 0.05 implied HWE compliance. Studies with NOS ≤ 5, studies with controls that deviated from HWE, and studies where not enough data were available for HWE calculation, were excluded from further analysis. Wherever possible, the data were extracted separately for different nsOFC phenotypes, with division into three groups: nsOFC, as all phenotypes together; nsCL/P, as cleft lip with or without cleft palate; and nsCPO, as cleft palate only. Where the phenotype was not clearly defined, the assumption was that all nsOFC subtypes were combined.

The associations between most of the genetic markers and nsOFC were evaluated as ORs with 95% CIs, based on four distinct genetic models: allelic model (minor allele [a] vs. major allele [A]); dominant model (Aa + aa vs. AA); recessive model (aa vs. Aa + AA); and overdominant model (Aa vs. AA + aa). The studies that did not provide genotype counts of the polymorphisms studied were included in the allelic model analysis, but not in the analysis with the three other genetic models.

To evaluate between-study heterogeneity, Cochrane Q tests and the I^2^ statistic were used. If I^2^ < 50% or if the Q test p > 0.1, the assumption was for no significant heterogeneity and a fixed-effects model was used to pool the ORs. Otherwise, a random-effects model was applied. The significance of the results (i.e., the pooled ORs) was determined using Z-tests, with p ≤ 0.05 considered statistically significant. In addition, a Bonferroni correction was applied to adjust for multiple tests due to four different genetic models (i.e., allelic, dominant, recessive, overdominant) and three different phenotypes (i.e., nsOFC, nsCL/P, nsCPO), which resulted in 12 multiple tests in total and a Bonferroni-corrected threshold of 0.0042. For genetic markers not having all 12 multiple tests performed (due to, e.g., no data on genotype counts or specific phenotype), an actual number of multiple tests performed was used, which equals to the product of number of phenotypes assessed and number of genetic models obtained. In such a case, a corrected threshold was recalculated accordingly. For example, four genetic models and two phenotypes resulted in eight multiple tests per genetic marker, and thus, a corrected threshold of 0.0063.

The pooled ORs represented the effects size of a genetic marker for each nsOFC phenotype and genetic model used. The significant effects sizes were divided into three categories based on the magnitudes: large (OR ≤ 0.50; OR ≥ 2.00), moderate (0.50 < OR ≤ 0.83; 1.20 ≤ OR < 2.00), and small (0.83 < OR < 1.20). The lower threshold value (1.20 or 1/1.20 [i.e., 0.83]) was set from Ioannidis et al., which indicates that effect sizes of individual genetic markers for complex diseases < 1.2 are possible, but very difficult to differentiate for the potential impact of bias^31^. The upper threshold value (2.00 or 0.50) was arbitrarily set, which indicates doubled odds (or risks) associated with nsOFC if a specific genetic marker is present.

All of the statistical analyses were carried out using Microsoft Excel (2016) or Review Manager (RevMan) version 5.4.1 (The Cochrane Collaboration Software, 2020).

## Results and discussion

### Study selection

**Figure 1:**
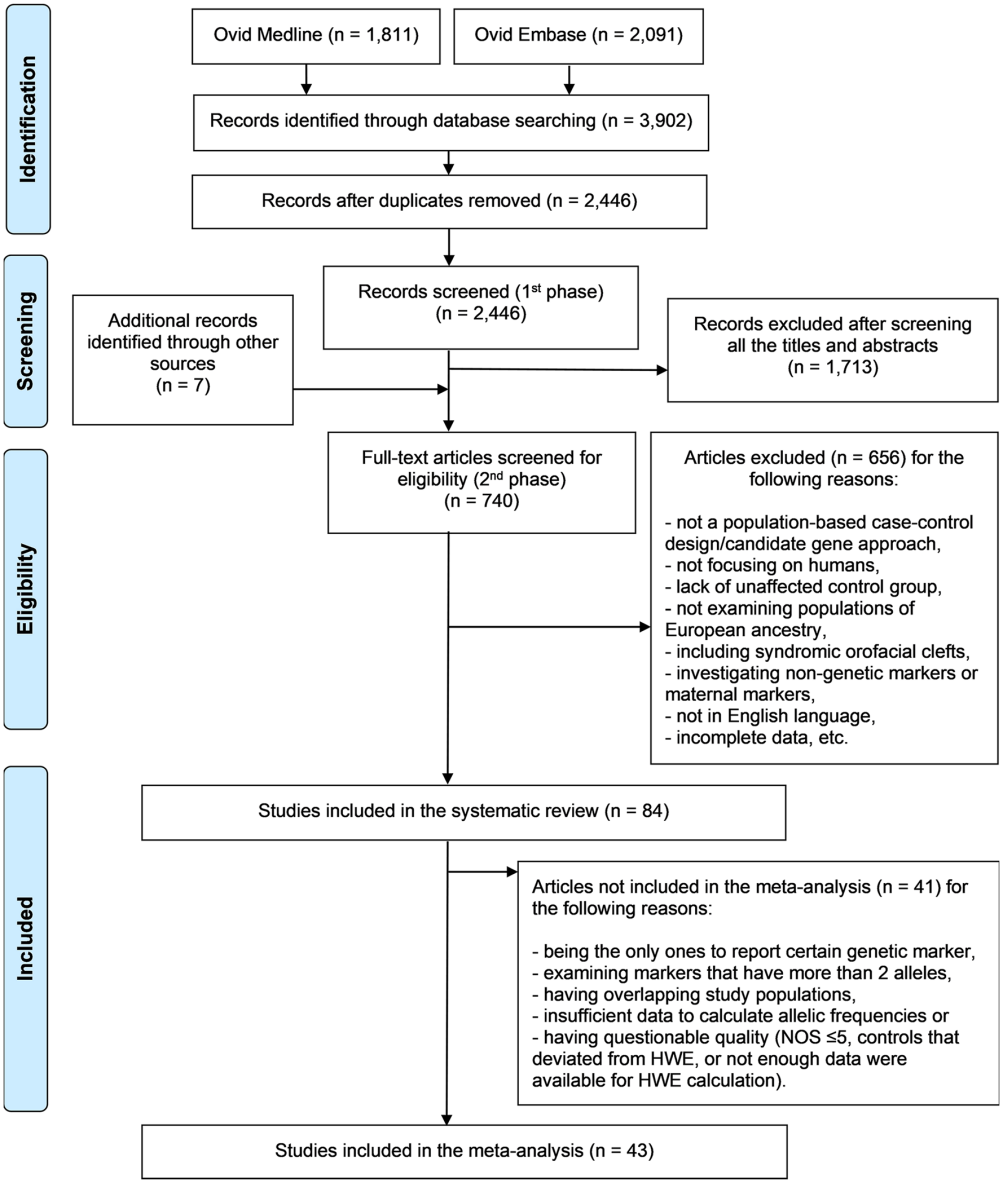
Flowchart of study identification and inclusion.

The detailed processes of the study identification and inclusion are summarised in the flowchart in Fig. 1. The search strategy used identified a total of 3902 studies. After removal of duplicate studies, 2446 studies remained. The first phase of selection (i.e., based on titles and abstracts) led to exclusion of 1713 studies, so leaving 733 studies. During the screening process, seven additional studies were identified. Therefore, a total of 740 potentially relevant studies were included in the second phase (i.e., based on full text) for evaluation based on the full eligibility criteria. Finally, after exclusion of 656 studies, 84 studies remained eligible for inclusion in the systematic review, 43^10–12,32–71^ of which were also included in the meta-analysis.

### Quality assessment of studies included in the systematic review

The NOS scores of the studies included in the systematic review ranged from 4 to 9, with a mean of 7.1. This indicates that the majority of the studies were of good or high methodological quality. Only five studies (of 84; 6.0%) had NOS ≤ 5 (i.e., four had NOS 5 and one had NOS 4). None of these five studies was included in the meta-analysis. The potential risk of bias in the selected studies mainly resulted from improper selection and definition of controls and non-comparability between cases and controls (based on gender, age or geographic origin).

### Characteristics of studies included in the systematic review

The main characteristics of the 43 studies^10–12,32–71^ included in the meta-analysis are presented in Supplementary Table S1, with those of the 41 studies included in the systematic review but not in the meta-analysis in Supplementary Table S2. The articles were published between 1989 and 2019, and were all written in the English language. The 84 case–control studies examined European-ancestry samples coming mainly from Europe (i.e., Austria, Denmark, Estonia, France, Germany, Ireland, Italy, Latvia, Lithuania, The Netherlands, Norway, Poland, Slovakia, Slovenia, Ukraine, the United Kingdom), and also from North America (United States of America), South America (Brazil) and Australia.

**Figure 2:**
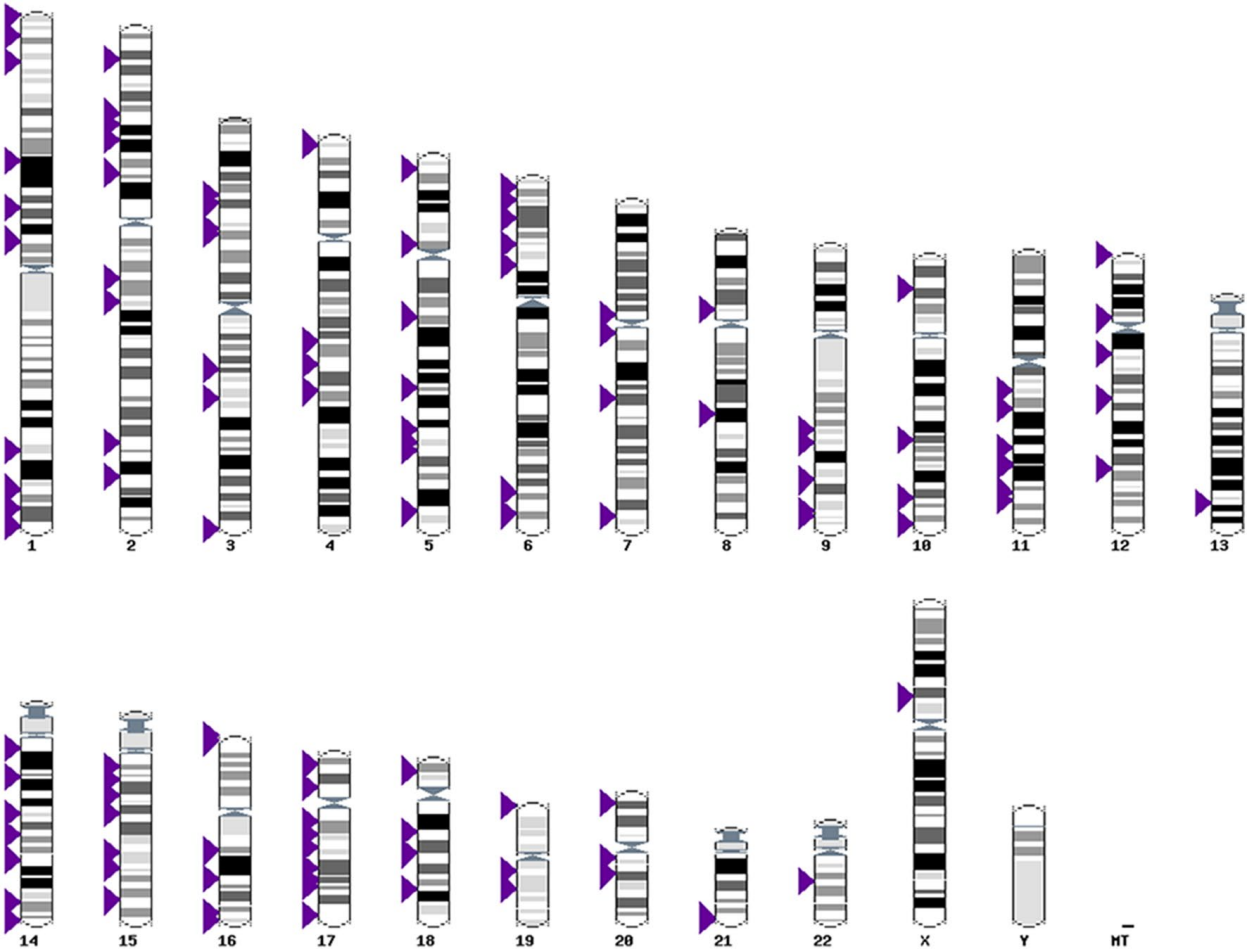
Karyotype display for all of the genetic loci analysed for non-syndromic orofacial cleft in the populations of European ancestry (purple arrowheads) that were extracted from the 84 studies selected. Data are presented using Ensembl genomic browser, release 101^72^.

The 84 case–control studies included in the systematic review examined associations of > 700 genetic markers with nsOFC in these populations of European ancestry. These markers were distributed throughout the genome (Fig. 2), and within many genes and non-coding regions. Supplementary Table S3 lists the genetic markers included in the systematic review but not in the meta-analysis, with their chromosome locations and their sources and references. A total of 47 genetic markers (out of > 700) were investigated in two or more studies.

### Overall results of the meta-analysis

The 43 studies included in the meta-analysis^10–12,32–71^ examined the effects of genetic variants on the development of the different nsOFC phenotypes. Although some of the studies investigated all of the nsOFC subtypes together (n = 9; 20.9%), the majority (n = 34; 79.1%) studied nsCL/P and/or nsCPO separately. While nsCL/P was the phenotype of choice in almost all of the studies with the separate phenotypes (n = 32; 94.1%), the nsCPO cases were assessed in only 52.9% (n = 18). Furthermore, the size of the nsCPO sample was smaller than the nsCL/P sample in all of the studies that included both phenotypes (Supplementary Tables S1, S2). Using the different genetic models, the majority of the 43 studies showed associations between at least one of the genetic markers studied and one of the nsOFC subtypes.

Meta-analysis was performed for 47 genetic markers in or in close proximity to 29 genes, and in 1 non-coding region (8q24). These were genes that encode transcription factors (*IRF6*, *GRHL3, VAX1*, *FOXE1*, *MAFB*), growth factors or receptors of growth factors (*TGFA*, *TGFB3*, *FGF10*, *FGFR1*), cell adhesion proteins (*CDH1*), transporters (*ABCA4*), enzymes (*MMP9*), and proteins involved in cellular signalling (*NOG*, *GREM1*, *WNT3*, *WNT3A*, *WNT5A*, *WNT8A*, *WNT9B*, *DVL2*, *AXIN2*, *APC*, *CTNNB1*) and in metabolism of nutrients (*MTHFR*, *MTHFD1*, *MTR*, *MTRR*, *BHMT*, *TCN2*). The abbreviations of the gene names are provided in Supplementary Table S4. In some of the studies, the genotype frequencies were not reported; therefore, the calculations for the dominant, recessive and overdominant models were not possible.

For most of the genetic markers (n = 27; 57.4%), enough data were available such that the analysis was carried out not only for pooled nsOFC phenotypes, but also separately for the nsCL/P subtype. In contrast, the nsCPO phenotype was only evaluated for a total of five genetic markers. For some of the markers, only one type of phenotype was analysed: nsCL/P for 13 markers; nsCPO for 1 marker; or all phenotypes combined as nsOFC for 6 markers. More than half of the markers were studied in only two eligible studies. The complete results of the meta-analysis are given in the Tables 1, 2, Supplementary Tables S5 and S6. The forest plots for the allelic genetic models are given in Supplementary Figs. S1–S47, and the forest plots of the other genetic models (i.e., dominant, recessive, overdominant) are available upon reasonable request to the corresponding author.

**Table 1:** Results of the meta-analysis performed to determine the associations between the genetic markers and the different non-syndromic orofacial cleft phenotypes in populations of European ancestry; results for the genes where there were significant associations with large effect sizes.

Gene	Variant^a^	A/a	Phenotype	N	N_CA_	N_CO_	Allelic model^b^		Dominant model^c^		Recessive model^d^		Overdominant model^e^	
							OR (95% CI)	p-value	OR (95% CI)	p-value	OR (95% CI)	p-value	OR (95% CI)	p-value
GRHL3	rs41268753***	C/T	CPO	4	487	2223	**2.29 (1.68–3.11)**	** < 0.00001**	**2.70 (1.66–4.38)**	** < 0.0001**	NE	NE	**2.79 (1.70–4.56)**	** < 0.0001**
IRF6	rs642961**	G/A	OFC	4	873	2025	**1.56 (1.36–1.78)**	** < 0.00001**	**1.73 (1.47–2.04)**	** < 0.00001**	**1.58 (1.11–2.26)**	**0.012**^**#**^	**1.61 (1.36–1.90)**	** < 0.00001**
			CL/P	3	699	1699	**1.55 (1.34–1.79)**	** < 0.00001**	**1.75 (1.46–2.10)**	** < 0.00001**	1.55 (0.75**–**3.21)	0.237	**1.65 (1.37–1.98)**	** < 0.00001**
	rs2013162**	C/A	OFC	2	1185	1813	**0.89 (0.80–0.99)**	**0.039**^**#**^	0.88 (0.76**–**1.03)	0.104	0.78 (0.52**–**1.16)	0.218	0.97 (0.83**–**1.12)	0.663
			CL/P	2	907	1813	**0.84 (0.74–0.95)**	**0.004**	**0.84 (0.71–0.99)**	**0.032**^**#**^	**0.68 (0.52–0.90)**	**0.007**^**#**^	0.97 (0.82**–**1.13)	0.666
	rs2235371***	G/A	OFC	2	1187	1846	**0.41 (0.23–0.71)**	**0.001**	**0.38 (0.21–0.67)**	**0.001**	NE	NE	**0.35 (0.20–0.63)**	**0.0005**
			CL/P	2	902	1846	**0.35 (0.19–0.67)**	**0.001**	**0.35 (0.18–0.66)**	**0.001**	NE	NE	**0.35 (0.18–0.66)**	**0.001**
	rs590223	A/G	CL/P	2	275	372	1.19 (0.83**–**1.71)	0.355	NA	NA	NA	NA	NA	NA
TGFA	TaqI**	A1/A2	OFC	5	869	1139	1.45 (0.92**–**2.26)	0.108	1.63 (0.92**–**2.89)	0.091	0.73 (0.23**–**2.26)	0.580	1.30 (0.99**–**1.69)	0.056
			CL/P	5	596	1139	1.32 (0.81–2.14)	0.259	1.46 (0.78**–**2.74)	0.235	1.22 (0.11 – 13.4)	0.870	1.18 (0.87**–**1.60)	0.290
			CPO	4	247	1079	1.33 (0.97**–**1.82)	0.076	1.48 (0.99**–**2.20)	0.056	NE	NE	**1.50 (1.00–2.25)**	**0.050**^**#**^
	BamHI***	A2/A1	CL/P	2	134	156	0.55 (0.20**–**1.54)	0.253	**0.48 (0.26–0.89)**	**0.021**^**#**^	NE	NE	**0.43 (0.23–0.82)**	**0.010**
	RsaI	A2/A1	CL/P	2	137	160	1.10 (0.77**–**1.56)	0.600	1.30 (0.82**–**2.07)	0.261	0.79 (0.39**–**1.61)	0.514	1.47 (0.92**–**2.35)	0.111
8q24	rs987525***	C/A	OFC	5	1067	3110	**1.94 (1.60–2.34)**	** < 0.00001**	**2.01 (1.52–2.67)**	** < 0.00001**	**3.51 (2.66–4.64)**	** < 0.00001**	**1.50 (1.20–1.89)**	**0.0005**
			CL/P	4	844	2784	**2.20 (1.94–2.49)**	** < 0.00001**	**2.42 (2.06–2.85)**	** < 0.00001**	**4.01 (2.93–5.49)**	** < 0.00001**	**1.74 (1.47–2.04)**	** < 0.00001**
VAX1	rs7078160***	G/A	OFC	3	471	1023	**1.60 (1.31–1.94)**	** < 0.00001**	**1.58 (1.26–2.00)**	** < 0.0001**	**3.15 (1.70–5.83)**	**0.0003**	**1.36 (1.07–1.73)**	**0.011**^**#**^
			CL/P	2	306	697	**1.70 (1.34–2.16)**	** < 0.0001**	**1.64 (1.24–2.18)**	**0.001**	**4.30 (2.08–8.87)**	** < 0.0001**	1.33 (0.99**–**1.78)	0.057

A/a, major and minor alleles, where major allele is listed first and risk allele is underlined; Phenotype, non-syndromic orofacial cleft phenotype studied (CL/P, cleft lip with or without cleft palate; CPO, cleft palate only; OFC, all phenotypes combined); N, number of studies included in the analysis (allelic model); N_CA_, total number of cases included in the analysis (allelic model); N_CO_, total number of controls included in the analysis (allelic model); NA, not available; NE, not estimable; OR (95% CI), pooled odds ratio with 95% confidence interval for each analysis.

Abbreviations of gene names are given in Supplementary Table S4 and further details about the meta-analysis are presented in Supplementary Table S6.

*Significant results with large (***) and moderate (**) effect sizes; the largest values for the variant were considered, regardless of the phenotype studied or genetic model used.

^#^Results that did not remain significant after applying Bonferroni correction.

^a^Genetic variants found in named genes or in their close proximity; listed based on chromosomal location; ^b^minor allele (a) vs. major allele (A); ^c^Aa + aa vs. AA; ^d^aa vs. Aa + AA; ^e^Aa vs. AA + aa. Statistically significant results are in bold.

**Table 2:** Results of the meta-analysis performed to determine the associations between the genetic markers and the different non-syndromic orofacial cleft phenotypes in populations of European ancestry; results for the genes where there were significant associations with moderate effect sizes.

Gene	Variant^a^	A/a	Phenotype	N	N_CA_	N_CO_	Allelic model^b^		Dominant model^c^		Recessive model^d^		Overdominant model^e^	
							OR (95% CI)	p-value	OR (95% CI)	p-value	OR (95% CI)	p-value	OR (95% CI)	p-value
ABCA4	rs560426	G/A	CL/P	2	582	846	1.16 (0.70**–**1.94)	0.570	1.14 (0.66**–**1.97)	0.642	1.31 (0.58**–**2.96)	0.523	0.89 (0.72**–**1.11)	0.298
	rs481931**	C/A	CL/P	2	589	849	**0.78 (0.67–0.91)**	**0.002**	**0.74 (0.60–0.92)**	**0.008**	**0.70 (0.52–0.96)**	**0.025**^**#**^	0.92 (0.60**–**1.41)	0.709
WNT3a	rs708111**	T/C	OFC	2	673	547	1.16 (0.99**–**1.37)	0.068	NA	NA	NA	NA	NA	NA
			CL/P	2	582	547	**1.20 (1.02–1.42)**	**0.032**^**#**^	NA	NA	NA	NA	NA	NA
	rs752107	C/T	OFC	2	673	547	0.91 (0.76**–**1.08)	0.290	NA	NA	NA	NA	NA	NA
			CL/P	2	582	547	0.88 (0.74**–**1.06)	0.170	NA	NA	NA	NA	NA	NA
WNT5a	rs566926**	C/A	OFC	2	673	547	**1.24 (1.04–1.49)**	**0.020**	NA	NA	NA	NA	NA	NA
			CL/P	2	582	547	**1.28 (1.06–1.54)**	**0.010**	NA	NA	NA	NA	NA	NA
FOXE1	rs4460498**	C/T	OFC	2	1206	1302	**0.79 (0.71–0.89)**	**< 0.0001**	0.77 (0.55**–**1.08)	0.131	**0.78 (0.62–0.98)**	**0.034**^**#**^	**0.82 (0.70–0.96)**	**0.012**^**#**^
			CL/P	2	1060	1302	**0.77 (0.68–0.87)**	**< 0.0001**	0.76 (0.53**–**1.11)	0.153	**0.73 (0.57–0.93)**	**0.010**^**#**^	**0.82 (0.70–0.97)**	**0.019**^**#**^
	rs3758249**	G/A	OFC	2	1200	1301	**0.81 (0.72–0.91)**	**0.0004**	0.85 (0.52**–**1.40)	0.530	0.79 (0.63**–**1.00)	0.053	0.97 (0.58**–**1.61)	0.908
			CL/P	2	1055	1302	0.84 (0.65**–**1.08)	0.168	0.84 (0.50**–**1.42)	0.524	**0.74 (0.58–0.94)**	**0.016**^**#**^	0.97 (0.59**–**1.61)	0.919
GREM1	rs1258763**	A/G	CL/P	2	435	1203	**0.71 (0.60–0.84)**	**< 0.0001**	**0.60 (0.48–0.75)**	**< 0.00001**	0.78 (0.53**–**1.13)	0.186	**0.66 (0.53–0.82)**	**0.0002**
DVL2	rs35594616**	C/T	OFC	2	705	630	1.14 (0.97**–**1.33)	0.106	**1.46 (1.16–1.83)**	**0.001**	0.78 (0.39**–**1.55)	0.476	**1.58 (1.11–2.23)**	**0.010**
	rs2074222**	G/A	OFC	2	744	615	1.08 (0.92**–**1.27)	0.331	**1.29 (1.03–1.61)**	**0.026**^**#**^	0.74 (0.29**–**1.89)	0.533	1.42 (0.90**–**2.24)	0.134
	rs222836*	C/T	OFC	2	702	616	**1.18 (1.01–1.37)**	**0.038**^**#**^	1.38 (0.75**–**2.53)	0.296	1.15 (0.90**–**1.47)	0.277	1.13 (0.61**–**2.11)	0.692
NOG	rs227731**	A/C	OFC	3	471	1020	**1.38 (1.18–1.62)**	**< 0.0001**	**1.59 (1.24–2.03)**	**0.0002**	**1.47 (1.13–1.91)**	**0.004**	1.13 (0.91**–**1.41)	0.269
			CL/P	2	306	694	**1.33 (1.10–1.61)**	**0.003**	**1.63 (1.20–2.23)**	**0.002**	1.25 (0.75**–**2.06)	0.392	1.23 (0.94**–**1.61)	0.137
AXIN2	rs2240308**	G/A	OFC	2	369	513	0.94 (0.77**–**1.13)	0.505	0.75 (0.55**–**1.02)	0.067	1.13 (0.82**–**1.56)	0.452	**0.74 (0.56–0.97)**	**0.027**^**#**^
			CL/P	2	359	513	0.94 (0.77**–**1.14)	0.525	0.74 (0.54**–**1.01)	0.057	1.15 (0.83**–**1.59)	0.392	**0.72 (0.55–0.95)**	**0.020**^**#**^

A/a, major and minor alleles, where major allele is listed first and risk allele is underlined; Phenotype, non-syndromic orofacial cleft phenotype studied (CL/P, cleft lip with or without cleft palate; CPO, cleft palate only; OFC, all phenotypes combined); N, number of studies included in the analysis (allelic model); N_CA_, total number of cases included in the analysis (allelic model); N_CO_, total number of controls included in the analysis (allelic model); NA, not available; OR (95% CI), pooled odds ratio with 95% confidence interval for each analysis.

Abbreviations of gene names are given in Supplementary Table S4 and further details about the meta-analysis are presented in Supplementary Table S6.

*Significant results with moderate (**) and small (*) effect sizes; the largest values for the variant were considered, regardless of the phenotype studied or genetic model used.

^#^Results that did not remain significant after applying Bonferroni correction.

^a^Genetic variants found in named genes or in their close proximity; listed based on chromosomal location; ^b^minor allele (a) vs. major allele (A); ^c^Aa + aa vs. AA; ^d^aa vs. Aa + AA; ^e^Aa vs. AA + aa. Statistically significant results are in bold.

### Detailed results of the meta-analysis, with discussion

Statistically significant associations were observed between at least one of the nsOFC phenotypes and 19 genetic variants, under at least one of the genetic models. These variants were located in or in close proximity to 1 non-coding region (8q24) and 12 genes that encode transcription factors (*IRF6*, *GRHL3*, *VAX1*, *FOXE1*), a growth factor (*TGFA*), a transporter (*ABCA4*) and proteins involved in cellular signalling (*NOG*, *GREM1*, *AXIN2*, *DVL2*, *WNT3A*, *WNT5A*). After applying Bonferroni correction, the association remained significant for 14 genetic variants in 1 non-coding region (8q24) and 10 genes (Tables 1, 2). Two of the genes (i.e., *WNT3A* and *AXIN2*) did not remain significantly associated with nsOFC. Meta-analysis has shown associations between some of the genetic markers and nsOFC previously^23–26^. However, to the best of our knowledge, this is the first study that provides meta-analysis of all of the genetic markers that have been repeatedly studied in association with nsOFC in populations of European ancestry.

The statistically significant data were classified based on the size of the pooled ORs (large, moderate, small effect sizes). For each genetic variant, the largest values were considered, regardless of the genetic model used or phenotype studied. When more than one variant of a gene was analysed, the gene was classified based on the result of the variant with the largest effect size. None of the genes with statistically significant findings fell into the category of genes with small effect sizes.

#### Genes with large effect sizes

Statistically significant large effect sizes were estimated for rs2235371 (*IRF6*), rs41268753 (*GRHL3*), rs987525 (8q24), rs7078160 (*VAX1*) and BamHI (*TGFA*) (Table 1). After applying Bonferroni correction, these variants’ effects remained significant. The significant data for these variants were obtained mainly in the analysis where there was low between-study heterogeneity and fixed-effects models were used (Supplementary Table S6). In the following, we present the data for the genes where there were significant associations with large effect sizes, to provide a more detailed description of each gene, and to discuss the involvement of the individual variants of nsOFC.

##### IRF6

For rs2235371 in *IRF6*, there was significant association with nsOFC and nsCL/P. The effect sizes for the two phenotypes were similar, although a bit larger for nsCL/P under all of the models performed: allelic, dominant and overdominant. Minor allele A of rs2235371 was the protective allele against nsOFC and nsCL/P. Two other polymorphisms studied in or near *IRF6*, rs642961 and rs2013162, also showed significant associations with nsOFC and nsCL/P, but the effect sizes were only moderate or small. While minor allele A of rs642961 increased the risk of nsOFC and nsCL/P, minor allele A of rs2013162 decreased the risk. A fourth polymorphism near *IRF6* (rs590223) was analysed in the nsCL/P group, but the association was not statistically significant. This might be because the sample was much smaller in comparison to the sample sizes of the other markers studied. These data are given in Table 1 and Supplementary Figs. S6–S9.

Interferon regulatory factor 6 (*IRF6*) encodes a transcription factor. Mutations in *IRF6* were discovered to be the cause of two syndromic forms of orofacial clefts: Van der Woude syndrome (VWS1; OMIM 119300), and popliteal pterygium syndrome (PPS; OMIM 119500)^73^. This resulted in the hypothesis that hypomorphic mutations in this gene are associated with nsOFC.

Polymorphisms in or near *IRF6* were previously studied in five meta-analyses^17,20,26,74,75^. The polymorphism in *IRF6* that showed significant and the largest effects on nsOFC in the present study was rs2235371, which was investigated in three of the previous meta-analyses. Wang et al., Wattanawong et al. and Xia et al. showed that minor allele A of rs2235371 is the protective allele against nsOFC in populations of Asian descent^26,74,75^. Moreover, Wang et al. and Wattanawong et al. performed subgroup analyses that also included the association in White people. These two meta-analyses showed similar trends, but the associations were not significant^26,75^. In comparison to the present meta-analysis that included only two studies^33,57^, Wang et al. and Wattanawong et al. included four and five studies, respectively. The extra studies that they included did not satisfy the inclusion criteria for the present study, and so they were excluded. This was because the study populations were either not of European ancestry or were not clearly defined, or because the data provided were incomplete^26,75^. Furthermore, here we show significant data for all of the genetic models studied for nsOFC, and especially for nsCL/P. As the present nsOFC sample was mainly from nsCL/P, we believe that minor allele A of rs2235371 is associated with decreased risk of nsCL/P. However, more studies need to be carried out to resolve the discrepancies with the previous meta-analyses.

For the second variant in *IRF6*, rs2013162, the same two studies were included in our meta-analysis^33,57^, and the effects on nsOFC and nsCL/P appeared to be smaller in comparison to the first variant, rs2235371. Wattanawong et al. studied this variant in connection with nsCL/P in Asian and White populations, and only showed associations for White people. As for the first variant, the present study included fewer studies than Wattanawong et al. due to the stricter inclusion criteria here^26^. The present analysis provides further evidence that minor allele A of rs2013162 has protective effects against nsCL/P.

The third variant located near *IRF6*, rs642961, was also investigated in previous meta-analyses^17,20,26,75^. Assis Machado et al. examined the association in a Brazilian population, and showed that minor allele A of rs642961 was positively associated with increased risk of nsOFC under the allelic model^20^. A significant association with nsOFC was also shown in populations of Asian descent^17,26,75^. Lee et al. showed that the effect was larger in Asian populations than in non-Asian populations^17^. Two of these meta-analyses studied White people through subgroup analysis. While Wattanawong et al. included more studies in their analysis (five) than Wang et al. did (three), both of them showed significant associations of rs642961 with nsOFC. Even though the present meta-analysis on this marker pooled the data of four studies, these were not the same studies as included in Wattanawong et al.^26,75^. One of the studies was excluded here that did not define the population as White, and one additional study that was included was performed on a Slovak population^60^. Moreover, we excluded one study that did not calculate whether the controls were in HWE. Here, there were moderate effects of minor allele A (rs642961) on nsOFC and nsCL/P. Due to low between-study heterogeneity and evidence from previous meta-analyses, we believe that rs642961 is involved in the aetiopathogenesis of nsCL/P. The involvement of *IRF6* mutations in nsOFC has also been detected in cohorts that included European samples, in genome-wide linkage studies^76,77^ and GWAS^71,78–80^.

##### GRHL3

For rs41268753 in *GRHL3*, only the nsCPO phenotype was studied. Significant association was seen here for the allelic, dominant and overdominant models, where the minor allele T increased the risk of nsCPO. These data are given in Table 1 and Supplementary Fig. S3.

Grainyhead-like 3 (*GRHL3*) is one of the orthologues of *Drosophila* grainy head (grh) and encodes a transcription factor. The initial evidence of *GRHL3* involvement in OFC was when mutations were reported for patients with Van der Woude syndrome (VWS2; OMIM 606713), with an unusual CPO phenotype, rather than CL/P, which is typical of VWS1^81,82^. Later Leslie et al. performed GWAS on only nsCPO, and showed significant association with rs41268753 in *GRHL3*^68^. In the present meta-analysis, we combined their replication sample results from populations of European ancestry (US American, Danish, Norwegian) with results obtained by Mangold et al. on central European, Latvian and English nsCPO patients^42,68^, and we showed that minor allele T of rs41268753 had significant and large effects on nsCPO. Importantly, this marker was only studied in a case–control study in a mixed Brazilian population, where a similar trend was recorded, although the association was not significant^83^. This implies that this nsCPO marker might be ethnically specific for populations of European origin. Of note, this variant was absent in a Chinese population^84^, which further supports this possibility.

##### 8q24 locus

Large effect sizes were also estimated for the association between rs987525 in 8q24 and both nsOFC and nsCL/P. The association was a bit stronger between the marker and nsCL/P. It was significant for all four of the genetic models, with minor allele A increasing the risk of nsOFC and nsCL/P. However, these data indicate that the association was the strongest under the recessive model. These data are given in Table 1 and Supplementary Fig. S25.

The 8q24 locus is an intergenic region, or ‘gene desert’. Birnbaum et al. were the first to identify an association between this locus and nsOFC, in GWAS^71^, and the locus was later independently identified in other GWAS and meta-analyses of GWAS^78–80,85,86^. In the 8q24 locus, rs987525 has been the most extensively studied polymorphism in association with nsOFC. A case–control meta-analysis on a Brazilian population showed significant association of rs987525 with nsOFC under allelic, heterozygous and homozygous models^20^. Moreover, two meta-analyses performed by Wattanawong et al. and Wang et al. examined rs987525 in White people as well as in other populations^26,75^. In the more recent study of Wattanawong et al., they showed significant association in White, Asian, and in mixed populations, although the effects of rs987525 were the strongest in White people^26^. We examined rs987525 in the meta-analysis with the same studies as Wattanawong et al., although we excluded one GWAS^86^ that did not have a replication phase, and we included another study performed on a Slovak population^60^. With the present analysis, there was also association under all of the models studied, which further demonstrates that minor allele A of rs987525 is a significant risk factor for nsCL/P in populations of European ancestry.

##### VAX1

For rs7078160, which is located in close proximity to *VAX1*, the analysis was performed for nsOFC and nsCL/P. The association was a bit stronger for nsCL/P, but significant for both. While the effect size on nsCL/P was moderate under the allelic and dominant models, it was large under the recessive model. Minor allele A increased the risk of developing nsOFC and nsCL/P. These data are given in Table 1 and Supplementary Fig. S28.

Ventral anterior homeobox 1 (*VAX1*) encodes a transcription factor that contains a homeobox (DNA-binding) domain. *VAX1* was initially associated with orofacial clefts when markers in or near this gene approached significance in two GWAS^80,87^. The meta-analysis performed on a Brazilian population did not find significant association between rs7078160 near *VAX1* and nsOFC under the allelic model^20^. Li et al. performed a meta-analysis on rs7078160 and showed significant association between minor allele A and nsOFC. Although they also performed stratified analysis by ethnicity and showed significant association in White people, there was between-study heterogeneity^24^. In the present meta-analysis, one of their studies was excluded, as it only reported on case–control results from GWAS^87^, while one additional study was also included^60^. We also performed stratified meta-analysis by phenotype (i.e., nsOFC, nsCL/P), where there was significant association under all of the genetic models performed. As we did not identify between-study heterogeneity and as the effect sizes were larger in the nsCL/P group, we believe that rs7078160 is associated with this phenotype.

##### TGFA

A large effect size was also noted for a variant in a BamHI restriction site in *TGFA*. Meta-analysis was performed here for the nsCL/P phenotype only and showed statistical significance under the dominant and overdominant models. In the *TGFA* gene, the RsaI and TaqI restriction sites were also examined. For the variant in the TaqI restriction site, all three phenotypes were studied (i.e., nsOFC, nsCL/P, nsCPO), but the association was significant only for nsCPO under the overdominant model. This association did not remain significant after applying Bonferroni correction. For the variant in the RsaI restriction site, it was only possible here to examine the association with nsCL/P, where we did not find any statistically significant associations. These data are given in Table 1 and Supplementary Figs. S13–S15.

Transforming growth factor A (*TGFA*) encodes a growth factor. Three meta-analyses assessed the variant in the TaqI restriction site of *TGFA* in different populations. While Lu et al., Yan et al. and Feng et al. included nine, ten and 14 studies on White people, respectively^23,88,89^, we only included five studies in the present meta-analysis. The reason was that some of their studies were excluded due to our strict criteria regarding the population characteristics and overlapping study populations, or because the studies did not provide adequate information. We also excluded two studies with NOS 5, and a study where insufficient data were available for HWE calculation. All the three previous meta-analyses showed significant associations with moderate effect sizes between the variant and nsOFC under the dominant model^23,88,89^. On the other hand, we included fewer studies and did not detect any significant association under the same model. Lu et al. and Yan et al. also investigated the association for nsCL/P and nsCPO in White people separately. Although both analyses showed association with nsCL/P under the dominant model, our analysis did not. Moreover, while Yan et al. did not report any association with nsCPO, Lu et al. reported significant association under the allelic and dominant models^23,88^. On the contrary, our results suggest significant association between the variant and nsCPO only under overdominant model, but the association did not remain significant after applying Bonferroni correction. Therefore, we believe that more studies with larger sample sizes should be performed to obtain more conclusive data.

Furthermore, Lu et al. also examined RsaI and BamHI restriction sites in White nsCL/P patients. For the variant in the RsaI restriction site in *TGFA*, we included the same two studies but excluded one study with NOS 5. As well as Lu et al., we did not show association between this variant and nsCL/P. Finally, for the variant in the BamHI restriction site in *TGFA*, the present study included the same studies as Lu et al. but again excluded one study with NOS 5. We showed significance and the largest effect size on nsCL/P under the dominant and overdominant models. Lu et al. detected significant association under the allelic and dominant models but did not perform the analysis under the overdominant model^23^. As the results of our analysis are consistent with previous meta-analyses, we believe that the variant in BamHI restriction site of *TGFA* is associated with nsCL/P in populations of European ancestry, while the variant in RsaI restriction site is not.

#### Genes with moderate effect sizes

For the following genes with statistically significant results for the pooled ORs, moderate effect sizes were seen: rs4460498 and rs3758249 (*FOXE1*); rs227731 (*NOG*); rs35594616 and rs2074222 (*DVL2*); rs2240308 (*AXIN2*); rs1258763 (*GREM1*); rs481931 (*ABCA4*); rs708111 (*WNT3a*); and rs566926 (*WNT5a*) (Table 2). After applying Bonferroni correction, the effect of rs708111 (*WNT3a*), rs2074222 (*DVL2*), and rs2240308 (*AXIN2*), did not remain significant. Along with significant variants with large effect sizes, significant data were seen for the listed variants mainly in the analysis where there was low between-study heterogeneity and fixed-effects models were used (Supplementary Table S6). Due to the space limitations, we present the data for the genes where there were significant associations with moderate effect sizes, and provide a more detailed description of each gene, with discussion of the involvement of the individual variants in nsOFC in Supplementary Discussion.

#### Statistically non-significant findings

For variants in or in close proximity to the following genes, there were no statistically significant associations with any of the three nsOFC phenotypes studied: *MTHFR* (rs1801131, rs1801133); *MTR* (rs1805087); *CTNNB1* (rs4533622); *MTRR* (rs1801394); *FGF10* (rs1448037); *BHMT* (rs3733890); *APC* (rs351771); *WNT8a* (rs2040862); *FGFR1* (rs328300, rs6987534); *MTHFD1* (rs2236225); *TGFB3* (rs3917200, rs2205181); *CDH1* (rs9929218); *WNT3* (rs12452064, rs9890413, rs111769); *WNT9B* (rs4968282, rs2165846); *MAFB* (rs13041247, rs11696257); *MMP9* (rs17576); and *TCN2* (rs1801198) (Supplementary Table S5, Supplementary Table S6). All of these genetic markers have small effect sizes.

### Limitations

The present study had several limitations that need to be addressed. Although we searched the two of the largest and most commonly used bibliographic databases (i.e., Ovid Medline and Ovid Embase), we did not search other databases (e.g., Scopus, Web of Science, grey literature databases). The reason lies in the fact that we did not focus on a limited number of genetic variants, but were looking for all genetic variants that have been studied in connection with nsOFC in populations of European ancestry. Thus, by searching the two mentioned databases, we identified a large number of studies. Due to that and our strict inclusion criteria, we found database search extremely time-consuming. Furthermore, all of the studies included in the systematic review were found in Ovid Medline, which means that we did not improve the quality of our search strategy by adding Ovid Embase. We realized that by adding additional bibliographic databases, we would not significantly contribute to the study. However, we acknowledge this as a limitation to our study.

Population-based case–control studies were included, but family-based association studies were omitted, which have often been used to examine connections between candidate genes and nsOFC.

In addition, although we reduced the heterogeneity among the individuals by limiting the population here to Europeans, it is still present to some extent, as we included studies/samples from different countries.

The data were also not adjusted for any other potential confounding factors. Moreover, we did not take into consideration gene–environment or gene–gene interactions, which can also be important in the aetiopathogenesis of this complex disorder. Some markers might well be associated with nsOFC through such interactions, but were not significantly associated in the present single marker meta-analysis.

Furthermore, due to the small number of studies that have examined associations between most of the markers and nsOFC in populations of European ancestry, it was not possible to evaluate publication bias in the present meta-analysis.

## Conclusions

In the present study, we systematically reviewed the available literature to identify 84 relevant population-based case–control studies where > 700 genetic markers were identified, to investigate their associations to nsOFC in a population of European ancestry. In this meta-analysis on repeatedly reported markers, statistically significant associations were seen between at least one of the nsOFC phenotypes and 19 genetic markers in or in close proximity to 12 genes and in 1 non-coding region. These data indicate that genetic variants in *IRF6*, *GRHL3*, 8q24, *VAX1*, *TGFA*, *FOXE1*, *ABCA4*, *NOG*, *GREM1*, *AXIN2*, *DVL2*, *WNT3A* and *WNT5A* are associated with nsOFC, nsCL/P or nsCPO under at least one of the genetic models included. To the best of our knowledge, this is the first meta-analysis to confirm the association between nsOFC and the following genetic variants in White people: *IRF6* (rs2235371); *GRHL3* (rs41268753); *FOXE1* (rs4460498, rs3758249); *ABCA4* (rs481931); *GREM1* (rs1258763); *DVL2* (rs35594616, rs2074222, rs222836); *AXIN2* (rs2240308); *WNT3a* (rs708111); and *WNT5a* (rs566926).

## Supplementary Information

Supplementary Information 1.

Supplementary Information 2.

Supplementary Information 3.

Supplementary Information 4.
