# Supplementary material for: Genetic markers for non-syndromic orofacial clefts in populations of European ancestry: a meta-analysis
Source: Sci Rep. 2022 Jan 24;12:1214. doi: 10.1038/s41598-021-02159-5 (PMC8786890; doi:10.1038/s41598-021-02159-5)
Supplement: Supplementary file 1 — Supplementary Information 1. [file 41598_2021_2159_MOESM1_ESM.docx]

**Supplementary Information**

**Supplementary Discussion**

**Genetic markers for non-syndromic orofacial clefts in populations of European ancestry: a meta-analysis**

Lara Slavec^1,2^, Nataša Karas Kuželički^2^, Igor Locatelli^3^, Ksenija Geršak^1,4*^

^1^ University Medical Centre Ljubljana, Division of Gynaecology and Obstetrics, Research Unit, Ljubljana, Slovenia

^2^ University of Ljubljana, Faculty of Pharmacy, Department of Clinical Biochemistry, Ljubljana, Slovenia

^3^ University of Ljubljana, Faculty of Pharmacy, Department of Social Pharmacy, Ljubljana, Slovenia

^4^ University of Ljubljana, Faculty of Medicine, Department of Gynaecology and Obstetrics, Ljubljana, Slovenia

***Corresponding author:**

Prof. Ksenija Geršak, MD, PhD

Tel: +386-1-522-6038

Email: [ksenija.gersak@mf.uni-lj.si](mailto:ksenija.gersak@mf.uni-lj.si)

**Supplementary Discussion**

FOXE1

For rs4460498 near *FOXE1*, the association with nsOFC and nsCL/P was tested, with significant association seen for both. The effect size was a bit larger for nsCL/P, where there was significant association under the allelic, recessive and overdominant models. Minor allele T had a protective effect against nsOFC and nsCL/P. For the second variant in *FOXE1*, rs3758249, significant results with moderate effect sizes were also obtained: for nsOFC under the allelic model, and for nsCL/P under the recessive model. Minor allele A was protective. These data are given in Table 2 and Supplementary Figures S26–S27.

Forkhead box E1 (*FOXE1*) encodes a transcription factor that contains a forkhead (DNA-binding) domain. *FOXE1* was initially connected to orofacial clefts when *FOXE1* mutations were reported to cause Bamforth–Lazarus syndrome (BLS; OMIM 241850), which includes cleft palate as part of the phenotype ^1,2^. Later, genome-wide linkage analyses ^3^ and meta-analyses of GWAS ^4^ reported significant association between mutations in or in close proximity to *FOXE1* and nsOFC. Two meta-analyses investigated association between rs4460498 and rs3758249 in or near *FOXE1* and nsOFC, but neither of them studied the effects on White patients separately ^5,6^. Two studies were included in all of the analyses performed here, which used fixed-effect models and showed significant association with moderate effect size between major allele C in rs4460498 and major allele G in rs3758249 and nsCL/P.

ABCA4

For variants in *ABCA4*, it was only possible here to examine the association with nsCL/P. The pooled ORs for rs481931 were significant under the allelic, dominant and recessive models, with major allele C increasing the risk. Surprisingly, no association was seen between another variant studied in *ABCA4* (rs560426) and nsCL/P. These data are given in Table 2 and Supplementary Figures S4–S5.

ATP binding cassette subfamily A member 4 (*ABCA4*) encodes an ABC transporter. Markers in or near *ABCA4* were initially associated with nsOFC in a GWAS, where case-parent trios from different populations were examined ^7^. While the association with rs560426 was significant in a replication cohort of families of European ancestry, there was no association with rs481931 ^7^. In the present study, we pooled the data from population-based case-control studies that investigated *ABCA4* with nsCL/P. In contrast to the GWAS replication results ^7^, here minor allele A in rs481931 was associated with decreased risk of nsCL/P, but there was no association with rs560426. Moreover, for rs560426, there was between-study heterogeneity. Based on the evidence from the present study, we believe that *ABCA4* has a role in nsCL/P. However, further large-scale population-based and family-based studies should be performed to confirm these findings.

NOG

There was significant association of rs227731 near *NOG* with nsOFC and nsCL/P under the allelic and dominant models, but not under the overdominant model. Minor allele C increased the risk of developing nsOFC and nsCL/P. The largest effect size was seen for nsCL/P under the dominant model, where otherwise these data for nsOFC and nsCL/P were similar. However, the effect sizes under the recessive model only showed significance for nsOFC, and thus not nsCL/P. The reason for this might be the larger number of studies included for nsOFC. These data are given in Table 2 and Supplementary Figure S42.

Noggin (*NOG*) encodes a signalling molecule. *NOG* was initially reported to be associated with nsOFC in a GWAS ^8^, and later in a GWAS meta-analysis ^9^. Two previous case-control meta-analyses were performed to examine the association between rs227731 near *NOG* and nsOFC. Interestingly, both of them showed that rs227731 is not associated with nsOFC in Asian populations. On the contrary, they showed significant association between rs227731 and nsOFC in White populations under the allelic, dominant and recessive models ^10,11^. However, the present meta-analysis included fewer studies, as some of those used previously were excluded due to not investigating populations of European ancestry, or to inclusion of GWAS data without any population-based case-control replication phase. We also identified one additional study. Nevertheless, significance was still seen here under the same models. These data suggest that minor allele C of rs227731 is involved in nsOFC in populations of European ancestry.

GREM1

For rs1258763, which is located in close proximity to *GREM1*, the association was only examined for nsCL/P. Here, there was significant association under the allelic, dominant and overdominant models, where major allele A increased the risk of nsCL/P. These data are given in Table 2 and Supplementary Figure S32.

Gremlin 1 (*GREM1*) encodes a signalling molecule. The locus 15q13 where *GREM1* is located was initially detected in association with nsCL/P in a GWAS ^8^, and reached genome-wide significance later in a subsequent GWAS meta-analysis ^12^. In the present study, rs1258763 near *GREM1* showed significant association under the allelic, dominant and overdominant models. These data suggest that minor allele G of rs1258763 has protective effects on nsCL/P in populations of European ancestry.

DVL2 and AXIN2

Significant data were also acquired for association between three variants in *DVL2* (rs35594616, rs2074222, rs222836) and nsOFC. For rs35594616, there was a moderate effect size under the dominant and overdominant models. On the other hand, rs2074222 only showed a moderate effect size under the dominant model, and rs222836 only showed a small effect size under the allelic model. After applying Bonferroni correction, the effect of rs35594616 remained significant, but the effects of rs2074222 and rs222836 did not. For rs2240308 in *AXIN2*, there was a moderate effect on nsOFC and nsCL/P under the overdominant model. These effects were similar, although a bit larger for nsOFC, probably due to the larger sample size. None of the effects remained significant after applying Bonferroni correction. These data are given in Table 2 and Supplementary Figures S34–S36, S43.

Dishevelled segment polarity protein 2 (*DVL2*) and Axin 2 (*AXIN2*) encode cell-signalling molecules. To the best of our knowledge, to date, there have been no meta-analyses for the association between *DVL2* or *AXIN2* markers and nsOFC. Here, we examined variants rs35594616, rs2074222, rs222836 in *DVL2* for association with nsOFC, and variant rs2240308 in *AXIN2* for association with nsOFC and nsCL/P. A case-control association study performed in an admixed Brazilian population also examined rs2074222, rs222836 and rs2240308 for association with nsCL/P, with significance only seen for rs2074222 (in *DVL2*) ^13^. The association of rs2240308 in *AXIN2* with nsCL/P was also shown in a family-based association study of cleft families from the USA ^14^. The present data suggest that both *DVL2* and *AXIN2* are associated with nsOFC in populations of European ancestry, although additional association studies and functional analyses are necessary to confirm these data.

WNT3A and WNT5A

In the present study, rs566926 in *WNT5a* showed positive association with nsOFC and nsCL/P, with the pooled OR greater for nsCL/P. In both instances, there were moderate effect sizes, where minor allele A increased the risk of developing nsOFC and nsCL/P. Interestingly, for rs708111 near *WNT3a*, there was also significant correlation with a moderate effect size between minor allele C and nsCL/P, but not for nsOFC. However, the effect of the variant did not remain significant after applying Bonferroni correction. For rs752107 in *WNT3a*, no significant associations were seen. These data are given in Table 2 and Supplementary Figures S10–S11, S17.

Wnt family member 3A (*WNT3A*) and Wnt family member 5A (*WNT5A*) are two further genes that encode cell-signalling molecules. These variants included in the present meta-analysis were previously studied in different populations, although without any meta-analyses. In a case-control study in a Chinese population, *WNT3A* markers (including rs752107) were significantly associated with nsOFC ^15^, as also in a family-based study on European Americans ^16^. In contrast, in the present study, there was association between nsCL/P and minor allele C in rs708111, but not with alleles in rs752107. Our data suggest that *WNT3A* is involved in nsCL/P in populations of European descent, although further association studies should be conducted to better support this conclusion.

Mutations in *WNT5A* have also been associated with Robinow syndrome (DRS1; OMIM 180700), where the phenotype sometimes involves orofacial clefts ^17^. Also, again in the family-based study on European Americans, a variant in *WNT5A* (rs566926) was associated with nsCL/P ^16^. In the present meta-analysis, rs566926 in *WNT5A* showed positive association with nsOFC and nsCL/P under the allelic model, which indicates that minor allele A represents the risk allele for nsOFC and nsCL/P. We believe that *WNT5A* is involved in nsOFC.

A meta-analysis by Wang et al. (2018) reported significant association between two polymorphisms of another gene in the Wnt signalling pathway, Wnt family member 3 (*WNT3*), and nsOFC ^18^. In contrast, in the present study, there was no association between markers in or near *WNT3* for nsOFC, as also seen with all of the other genes tested here for this pathway (i.e., *WNT8a*, *WNT9B*, *APC*, *CTNNB1*).

**References of Supplementary Discussion**

1. Bamforth, J. S., Hughes, I. A., Lazarus, J. H., Weaver, C. M. & Harper, P. S. Congenital hypothyroidism, spiky hair, and cleft palate. *J. Med. Genet.* **26**, 49 LP – 51; https://doi.org/10.1136/jmg.26.1.49 (1989).

2. Clifton-Bligh, R. J. *et al.* Mutation of the gene encoding human TTF-2 associated with thyroid agenesis, cleft palate and choanal atresia. *Nat. Genet.* **19**, 399–401; https://doi.org/10.1038/1294 (1998).

3. Marazita, M. L. *et al.* Meta-analysis of 13 genome scans reveals multiple cleft lip/palate genes with novel loci on 9q21 and 2q32-35. *Am. J. Hum. Genet.* **75**, 161–173; https://doi.org/10.1086/422475 (2004).

4. Leslie, E. J. *et al.* Genome-wide meta-analyses of nonsyndromic orofacial clefts identify novel associations between FOXE1 and all orofacial clefts, and TP63 and cleft lip with or without cleft palate. *Hum. Genet.* **136**, 275–286; https://doi.org/10.1007/s00439-016-1754-7 (2017).

5. Imani, M. M., Safaei, M., Lopez-Jornet, P. & Sadeghi, M. A systematic review and meta-analysis on protective role of forkhead box E1 (FOXE1) polymorphisms in susceptibility to non-syndromic cleft lip/palate. *Int. Orthod.* **17**, 437–445; https://doi.org/10.1016/j.ortho.2019.06.026 (2019).

6. Xiao, W., Jia, K., Yu, G. & Zhao, N. Association between forkhead box E1 polymorphisms and risk of non-syndromic cleft lip with or without cleft palate: a meta-analysis. *Orthod. Craniofac. Res.* **23**, 151–159; https://doi.org/10.1111/ocr.12366 (2020).

7. Beaty, T. H. *et al.* A genome-wide association study of cleft lip with and without cleft palate identifies risk variants near MAFB and ABCA4. *Nat. Genet.* **42**, 525–529; https://doi.org/10.1038/ng.580 (2010).

8. Mangold, E. *et al.* Genome-wide association study identifies two susceptibility loci for nonsyndromic cleft lip with or without cleft palate. *Nat. Genet.* **42**, 24–26; https://doi.org/10.1038/ng.506 (2010).

9. Ludwig, K. U. *et al.* Genome-wide meta-analyses of nonsyndromic cleft lip with or without cleft palate identify six new risk loci. *Nat. Genet.* **44**, 968–971; https://doi.org/10.1038/ng.2360 (2012).

10. Wang, Y. *et al.* Association between nonsyndromic cleft lip and palate and 2 polymorphic loci: a meta-analysis. *Cleft Palate-Craniofacial J.* https://doi.org/10.1177/1055665620962686 (2020).

11. Wang, F. *et al.* Associations between the NOGGIN rs227731 polymorphism and NSCL/P risk may be associated with ethnicities: a meta-analysis. *Birth defects Res.* **109**, 445–451; https://doi.org/10.1002/bdra.23612 (2017).

12. Ludwig, K. U. *et al.* Meta-analysis reveals genome-wide significance at 15q13 for nonsyndromic clefting of both the lip and the palate, and functional analyses implicate GREM1 as a plausible causative gene. *PLoS Genet.* **12**, e1005914; https://doi.org/10.1371/journal.pgen.1005914 (2016).

13. Araujo, T. K. de *et al.* A multicentric association study between 39 genes and nonsyndromic cleft lip and palate in a Brazilian population. *J. Cranio-Maxillofacial Surg.* **44**, 16–20; https://doi.org/10.1016/j.jcms.2015.07.026 (2016).

14. Letra, A. *et al.* Association of AXIN2 with non-syndromic oral clefts in multiple populations. *J. Dent. Res.* **91**, 473–478; https://doi.org/10.1177/0022034512440578 (2012).

15. Yao, T. *et al.* Association of Wnt3A gene variants with non-syndromic cleft lip with or without cleft palate in Chinese population. *Arch. Oral Biol.* **56**, 73–78; https://doi.org/10.1016/j.archoralbio.2010.09.002 (2011).

16. Chiquet, B. T. *et al.* Variation in WNT genes is associated with non-syndromic cleft lip with or without cleft palate. *Hum. Mol. Genet.* **17**, 2212–2218; https://doi.org/10.1093/hmg/ddn121 (2008).

17. Person, A. D. *et al.* WNT5A mutations in patients with autosomal dominant Robinow syndrome. *Dev. Dyn.* **239**, 327–337; https://doi.org/10.1002/dvdy.22156 (2010).

18. Wang, B.-Q. *et al.* Association of the WNT3 polymorphisms and non-syndromic cleft lip with or without cleft palate: evidence from a meta-analysis. *Biosci. Rep.* **38**, https://doi.org/10.1042/BSR20181676 (2018).
