# Supplementary material for: Genetic markers for non-syndromic orofacial clefts in populations of European ancestry: a meta-analysis
Source: Sci Rep. 2022 Jan 24;12:1214. doi: 10.1038/s41598-021-02159-5 (PMC8786890; doi:10.1038/s41598-021-02159-5)
Supplement: Supplementary file 2 — Supplementary Information 2. [file 41598_2021_2159_MOESM2_ESM.docx]

**Supplementary Information**

**Supplementary Figures S1–S47**

**Genetic markers for non-syndromic orofacial clefts in populations of European ancestry: a meta-analysis**

Lara Slavec^1,2^, Nataša Karas Kuželički^2^, Igor Locatelli^3^, Ksenija Geršak^1,4*^

^1^ University Medical Centre Ljubljana, Division of Gynaecology and Obstetrics, Research Unit, Ljubljana, Slovenia

^2^ University of Ljubljana, Faculty of Pharmacy, Department of Clinical Biochemistry, Ljubljana, Slovenia

^3^ University of Ljubljana, Faculty of Pharmacy, Department of Social Pharmacy, Ljubljana, Slovenia

^4^ University of Ljubljana, Faculty of Medicine, Department of Gynaecology and Obstetrics, Ljubljana, Slovenia

***Corresponding author:**

Prof. Ksenija Geršak, MD, PhD

Tel: +386-1-522-6038

Email: [ksenija.gersak@mf.uni-lj.si](mailto:ksenija.gersak@mf.uni-lj.si)

**A**


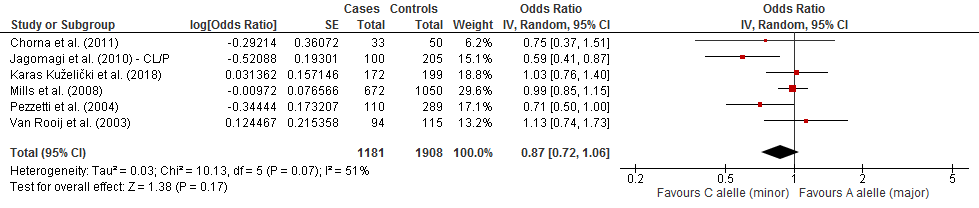


**B**


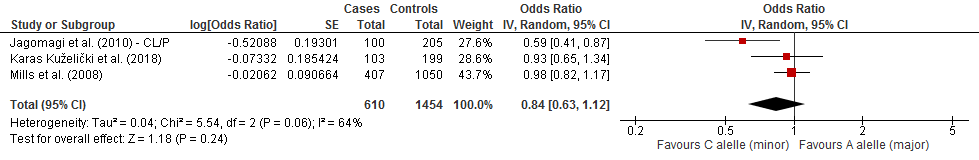


**C**


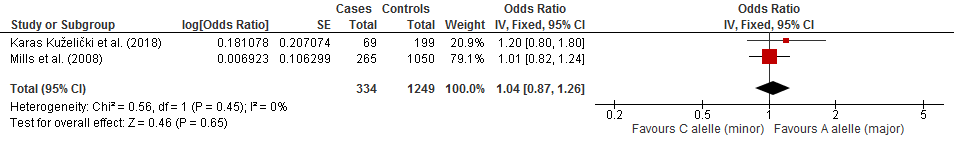


**Supplementary Figure S1.** Forest plots of the allelic genetic model analyses (minor allele vs. major allele) showing the association between rs1801131 in MTHFR and the different non-syndromic orofacial cleft phenotypes; (A), all phenotypes combined; (B), cleft lip with or without cleft palate; and (C), cleft palate only.

**A**

**
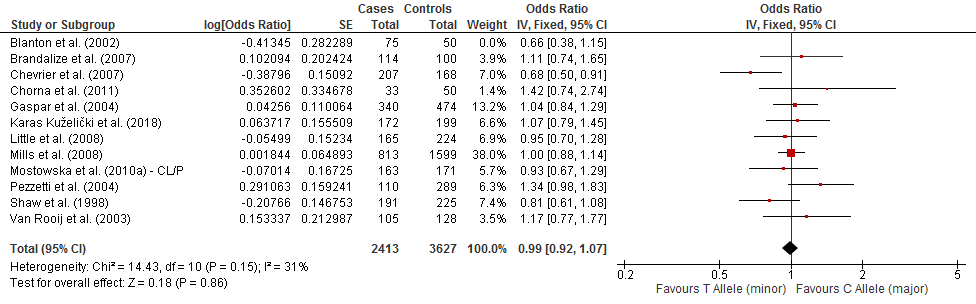
**

**B**


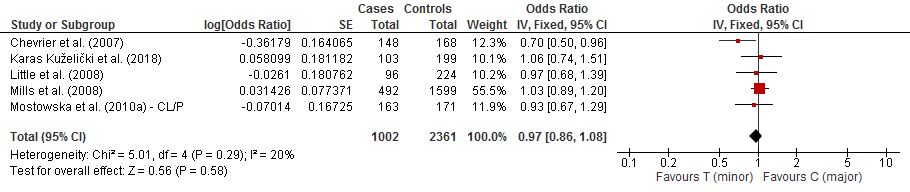


**C**


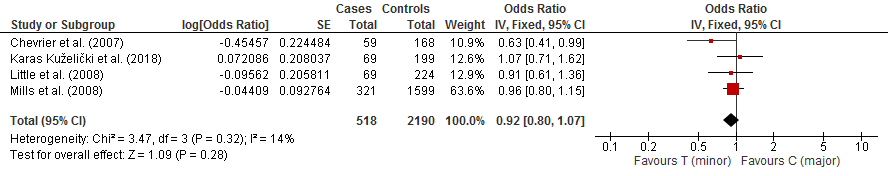


**Supplementary Figure S2.** Forest plots of the allelic genetic model analyses (minor allele vs. major allele) showing the association between rs1801133 in MTHFR and the different non-syndromic orofacial cleft phenotypes; (A), all phenotypes combined; (B), cleft lip with or without cleft palate; and (C), cleft palate only.


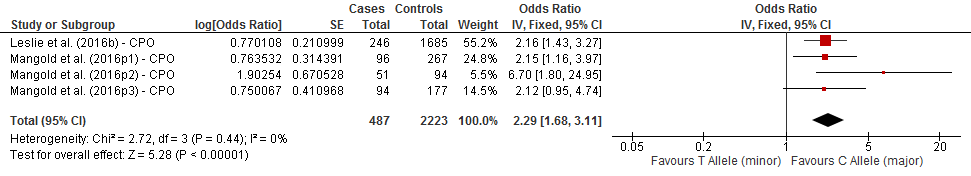


**Supplementary Figure S3.** Forest plot of the allelic genetic model analysis (minor allele vs. major allele) showing the association between rs41268753 in *GRHL3* and non-syndromic cleft palate only.


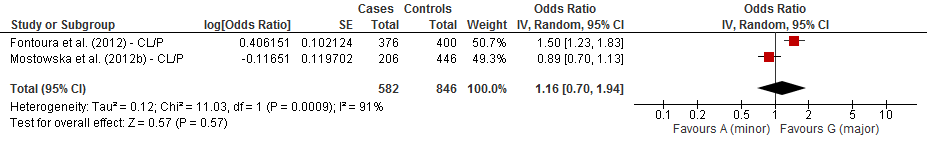


**Supplementary Figure S4.** Forest plot of the allelic genetic model analysis (minor allele vs. major allele) showing the association between rs560426 in *ABCA4* and non-syndromic cleft lip with or without cleft palate.


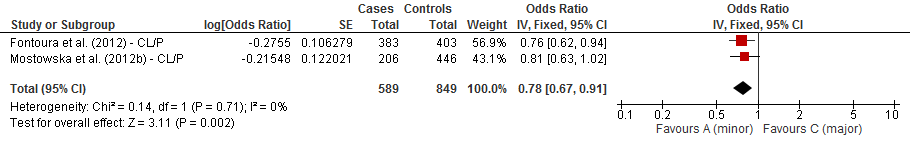


**Supplementary Figure S5.** Forest plot of the allelic genetic model analysis (minor allele vs. major allele) showing the association between rs481931 in *ABCA4* and non-syndromic cleft lip with or without cleft palate.

**A**


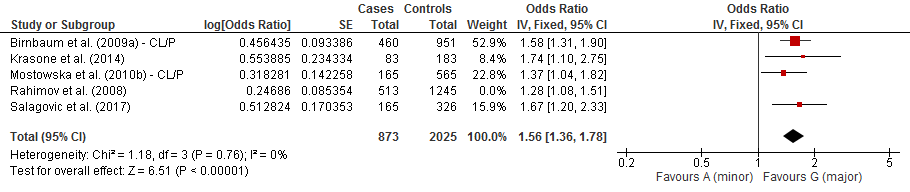


**B**


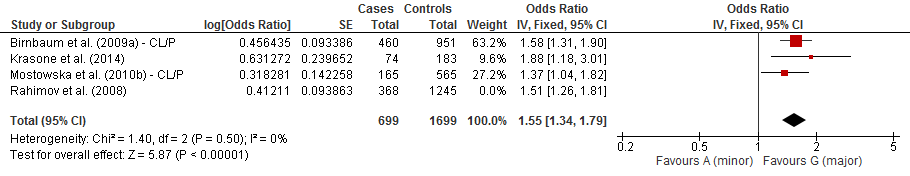


**Supplementary Figure S6.** Forest plots of the allelic genetic model analyses (minor allele vs. major allele) showing the association between rs642961 near *IRF6* and the different non-syndromic orofacial cleft phenotypes; (A), all phenotypes combined; (B), cleft lip with or without cleft palate.

**A**


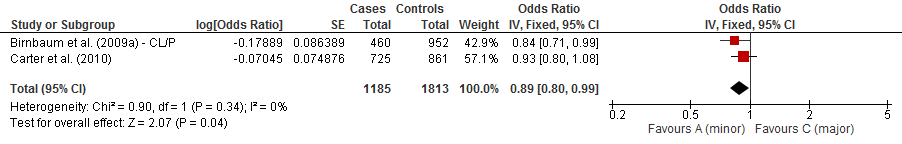


**B**


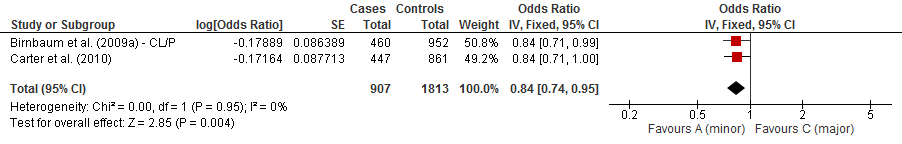


**Supplementary Figure S7.** Forest plots of the allelic genetic model analyses (minor allele vs. major allele) showing the association between rs2013162 in *IRF6* and the different non-syndromic orofacial cleft phenotypes; (A), all phenotypes combined; (B), cleft lip with or without cleft palate.

**A**


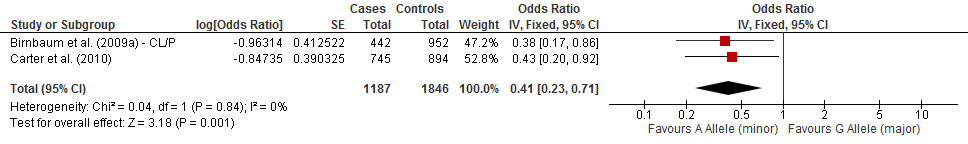


**B**


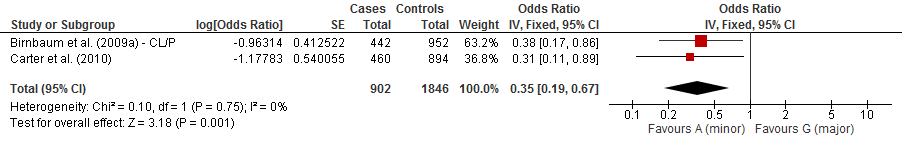


**Supplementary Figure S8.** Forest plots of the allelic genetic model analyses (minor allele vs. major allele) showing the association between rs2235371 in *IRF6* and the different non-syndromic orofacial cleft phenotypes; (A), all phenotypes combined; (B), cleft lip with or without cleft palate.


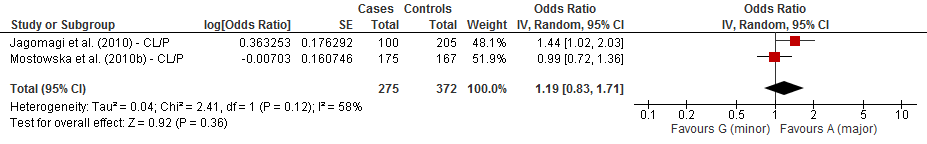


**Supplementary Figure S9.** Forest plot of the allelic genetic model analysis (minor allele vs. major allele) showing the association between rs590223 near *IRF6* and non-syndromic cleft lip with or without cleft palate.

**A**


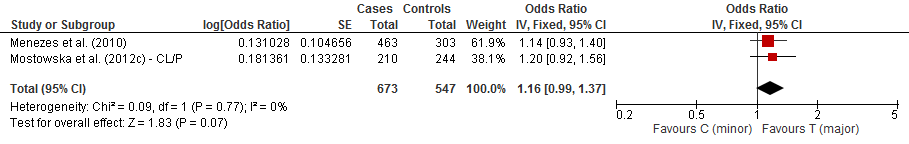


**B**


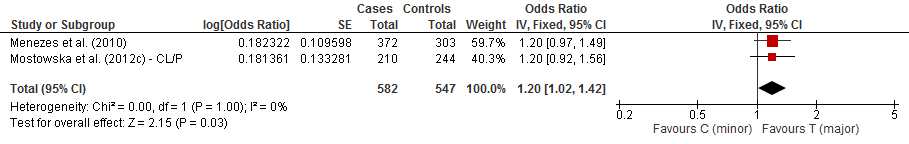


**Supplementary Figure S10.** Forest plots of the allelic genetic model analyses (minor allele vs. major allele) showing the association between rs708111 near *WNT3a* and the different non-syndromic orofacial cleft phenotypes; (A), all phenotypes combined; (B), cleft lip with or without cleft palate.

**A**


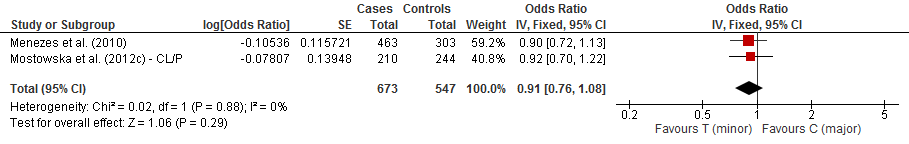


**B**


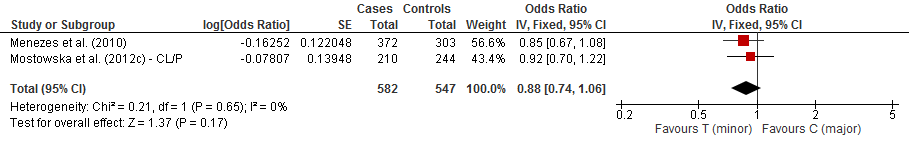


**Supplementary Figure S11.** Forest plots of the allelic genetic model analyses (minor allele vs. major allele) showing the association between rs752107 in *WNT3a* and the different non-syndromic orofacial cleft phenotypes; (A), all phenotypes combined; (B), cleft lip with or without cleft palate.


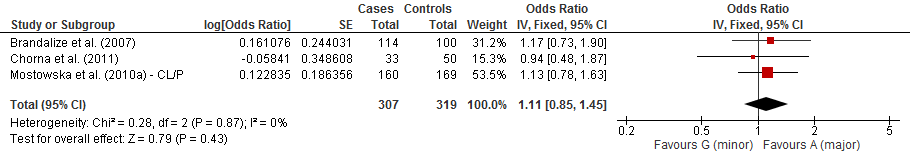


**Supplementary Figure S12.** Forest plot of the allelic genetic model analysis (minor allele vs. major allele) showing the association between rs1805087 in *MTR* and all different non-syndromic orofacial cleft phenotypes combined.

**A**


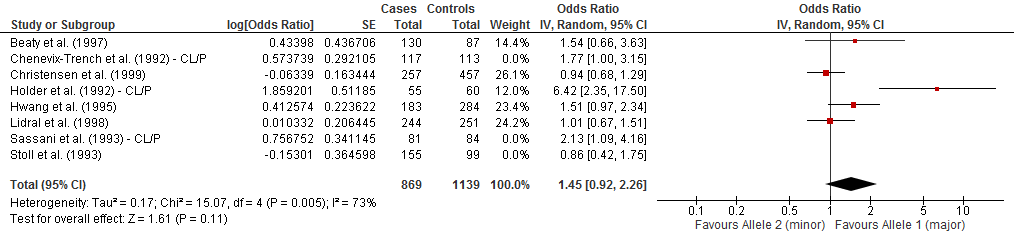


**B**


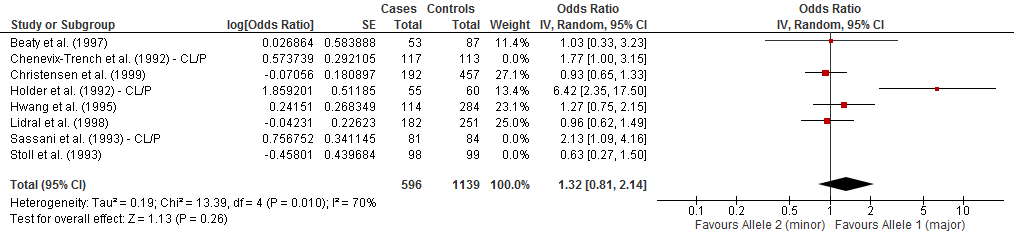


**C**


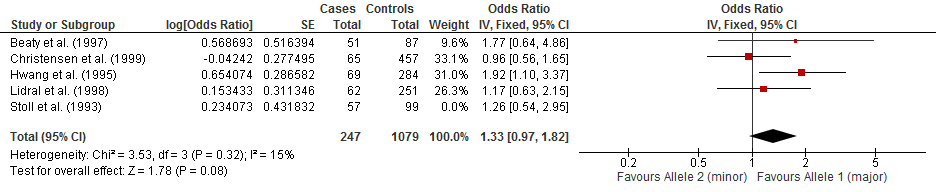


**Supplementary Figure S13.** Forest plots of the allelic genetic model analyses (minor allele vs. major allele) showing the association between TaqI in *TGFA* and the different non-syndromic orofacial cleft phenotypes; (A), all phenotypes combined; (B), cleft lip with or without cleft palate; and (C), cleft palate only.


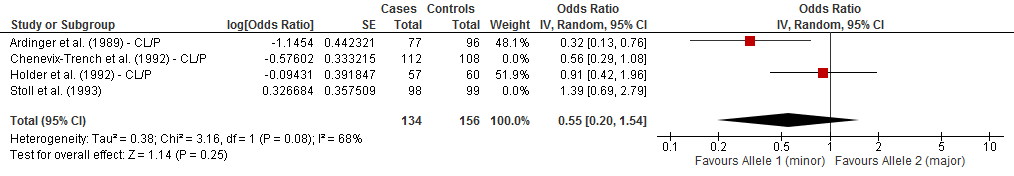


**Supplementary Figure S14.** Forest plot of the allelic genetic model analysis (minor allele vs. major allele) showing the association between BamHI in *TGFA* and non-syndromic cleft lip with or without cleft palate.


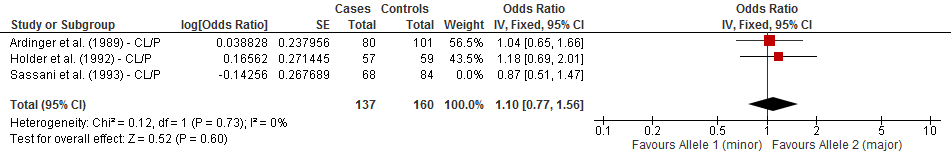


**Supplementary Figure S15.** Forest plot of the allelic genetic model analysis (minor allele vs. major allele) showing the association between RsaI in *TGFA* and non-syndromic cleft lip with or without cleft palate.


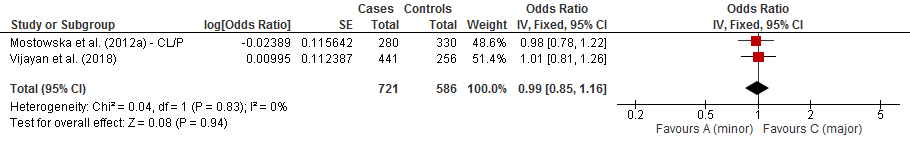


**Supplementary Figure S16.** Forest plot of the allelic genetic model analysis (minor allele vs. major allele) showing the association between rs4533622 in *CTNNB1* and all different non-syndromic orofacial cleft phenotypes combined.

**A**


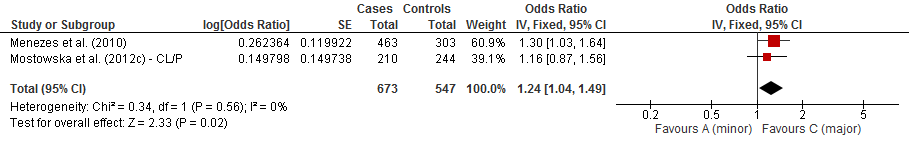


**B**


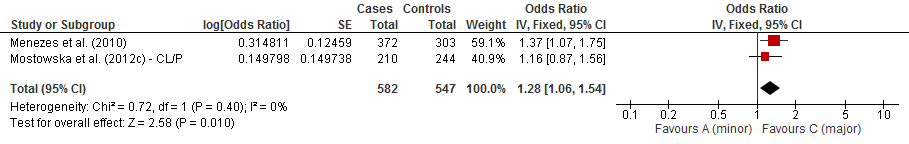


**Supplementary Figure S17.** Forest plots of the allelic genetic model analyses (minor allele vs. major allele) showing the association between rs566926 in *WNT5a* and the different non-syndromic orofacial cleft phenotypes; (A), all phenotypes combined; (B), cleft lip with or without cleft palate.

**A**


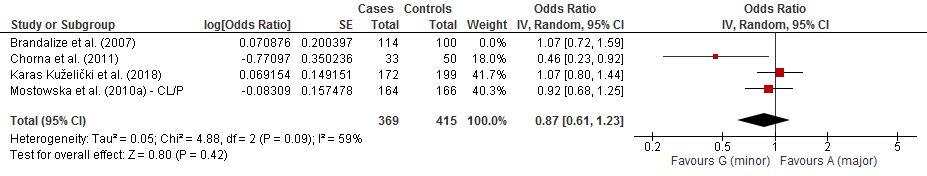


**B**


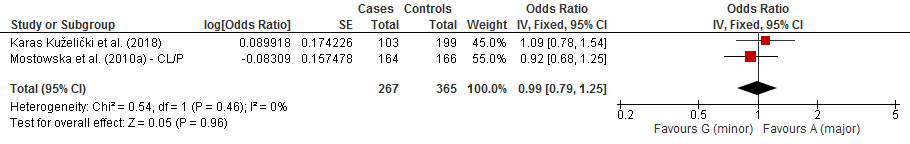


**Supplementary Figure S18.** Forest plots of the allelic genetic model analyses (minor allele vs. major allele) showing the association between rs1801394 in *MTRR* and the different non-syndromic orofacial cleft phenotypes; (A), all phenotypes combined; (B), cleft lip with or without cleft palate.

**A**


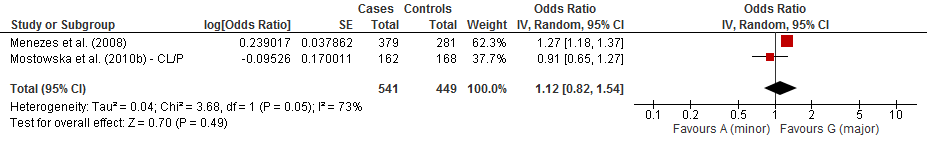


**B**


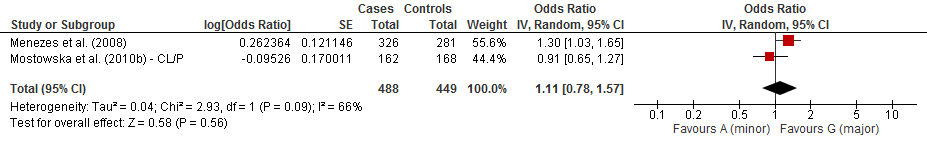


**Supplementary Figure S19.** Forest plots of the allelic genetic model analyses (minor allele vs. major allele) showing the association between rs1448037 in *FGF10* and the different non-syndromic orofacial cleft phenotypes; (A), all phenotypes combined; (B), cleft lip with or without cleft palate.

**A**


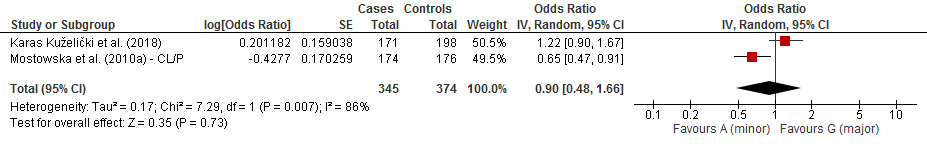


**B**


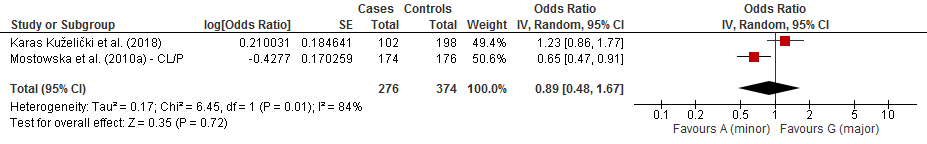


**Supplementary Figure S20.** Forest plots of the allelic genetic model analyses (minor allele vs. major allele) showing the association between rs3733890 in *BHMT* and the different non-syndromic orofacial cleft phenotypes; (A), all phenotypes combined; (B), cleft lip with or without cleft palate.


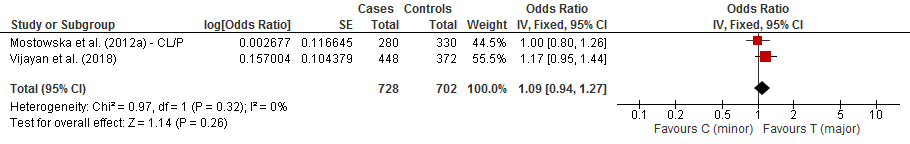


**Supplementary Figure S21.** Forest plot of the allelic genetic model analysis (minor allele vs. major allele) showing the association between rs351771 in *APC* and all different non-syndromic orofacial cleft phenotypes combined.

**A**


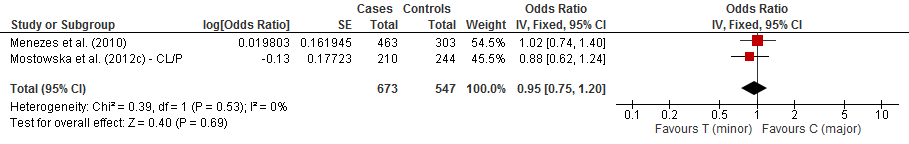


**B**


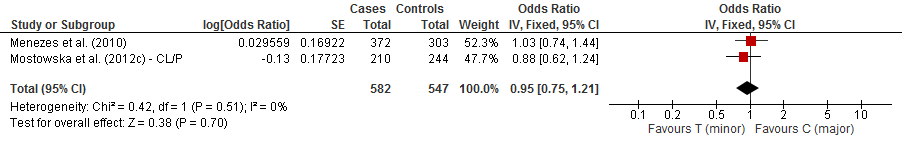


**Supplementary Figure S22.** Forest plots of the allelic genetic model analyses (minor allele vs. major allele) showing the association between rs2040862 in *WNT8a* and the different non-syndromic orofacial cleft phenotypes; (A), all phenotypes combined; (B), cleft lip with or without cleft palate.


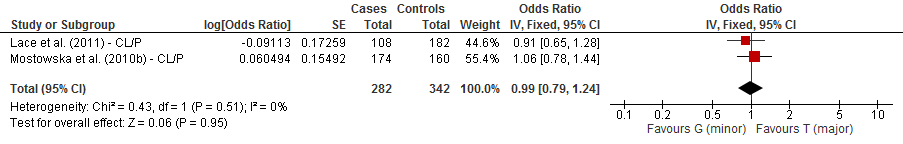


**Supplementary Figure S23.** Forest plot of the allelic genetic model analysis (minor allele vs. major allele) showing the association between rs328300 near *FGFR1* and non-syndromic cleft lip with or without cleft palate.


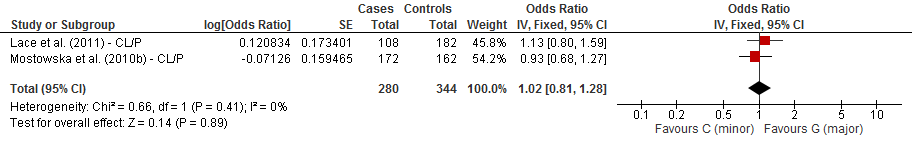


**Supplementary Figure S24.** Forest plot of the allelic genetic model analysis (minor allele vs. major allele) showing the association between rs6987534 in *FGFR1* and non-syndromic cleft lip with or without cleft palate.

**A**


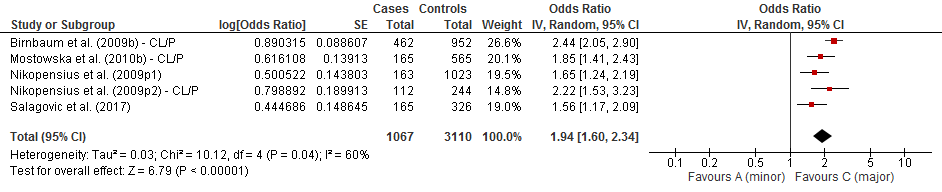


**B**


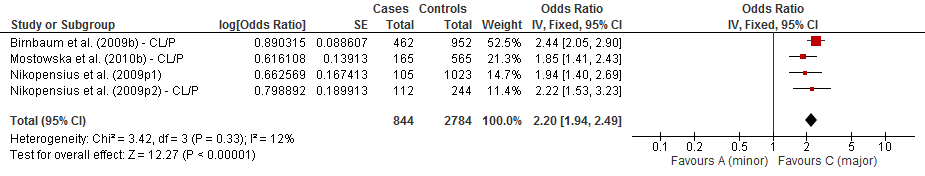


**Supplementary Figure S25.** Forest plots of the allelic genetic model analyses (minor allele vs. major allele) showing the association between rs987525 in 8q24 and the different non-syndromic orofacial cleft phenotypes; (A), all phenotypes combined; (B), cleft lip with or without cleft palate.

**A**


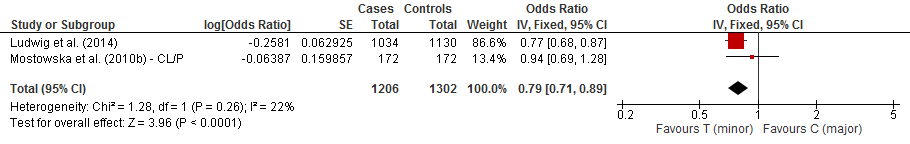


**B**


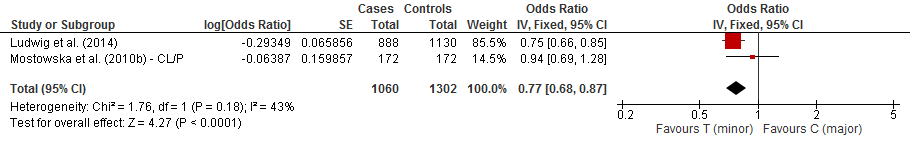


**Supplementary Figure S26.** Forest plots of the allelic genetic model analyses (minor allele vs. major allele) showing the association between rs4460498 near *FOXE1* and the different non-syndromic orofacial cleft phenotypes; (A), all phenotypes combined; (B), cleft lip with or without cleft palate.

**A**


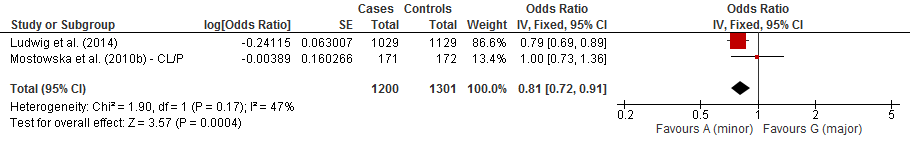


**B**


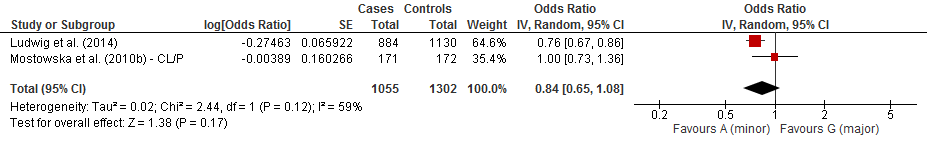


**Supplementary Figure S27.** Forest plots of the allelic genetic model analyses (minor allele vs. major allele) showing the association between rs3758249 in *FOXE1* and the different non-syndromic orofacial cleft phenotypes; (A), all phenotypes combined; (B), cleft lip with or without cleft palate.

**A**


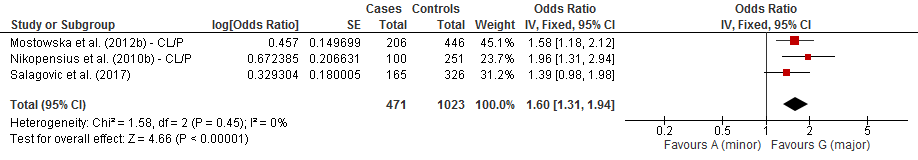


**B**


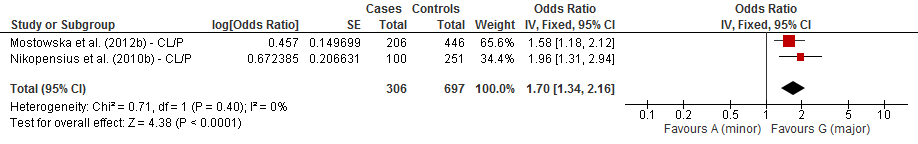


**Supplementary Figure S28.** Forest plots of the allelic genetic model analyses (minor allele vs. major allele) showing the association between rs7078160 near *VAX1* and the different non-syndromic orofacial cleft phenotypes; (A), all phenotypes combined; (B), cleft lip with or without cleft palate.

**A**


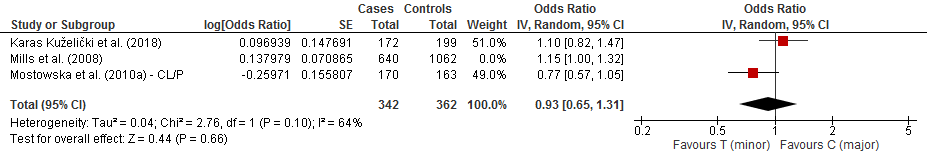


**B**


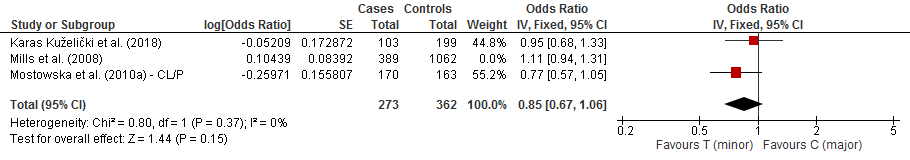


**Supplementary Figure S29.** Forest plots of the allelic genetic model analyses (minor allele vs. major allele) showing the association between rs2236225 in MTHFD1 and the different non-syndromic orofacial cleft phenotypes; (A), all phenotypes combined; (B), cleft lip with or without cleft palate.

**A**


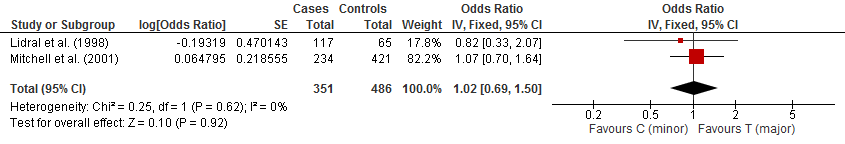


**B**


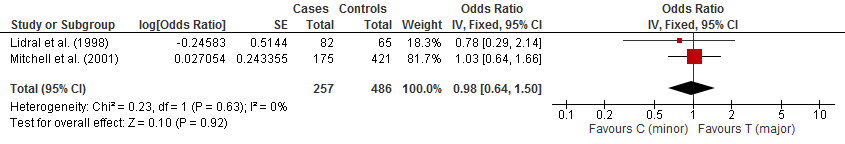


**C**


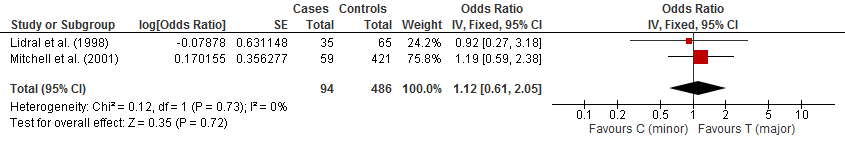


**Supplementary Figure S30.** Forest plots of the allelic genetic model analyses (minor allele vs. major allele) showing the association between rs3917200 in TGFB3 and the different non-syndromic orofacial cleft phenotypes; (A), all phenotypes combined; (B), cleft lip with or without cleft palate; and (C), cleft palate only.


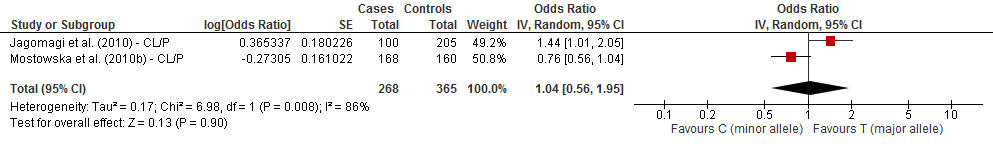


**Supplementary Figure S31.** Forest plot of the allelic genetic model analysis (minor allele vs. major allele) showing the association between rs2205181 near *TGFB3* and non-syndromic cleft lip with or without cleft palate.


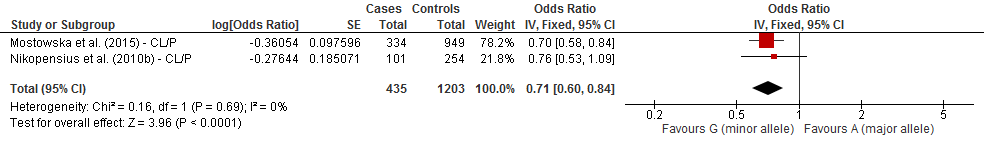


**Supplementary Figure S32.** Forest plot of the allelic genetic model analysis (minor allele vs. major allele) showing the association between rs1258763 near *GREM1* and non-syndromic cleft lip with or without cleft palate.

**A**


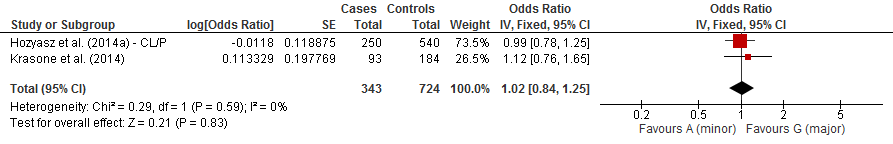


**B**


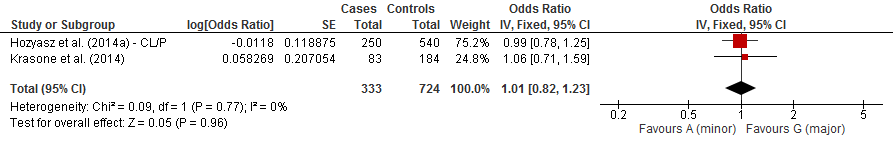


**Supplementary Figure S33.** Forest plots of the allelic genetic model analyses (minor allele vs. major allele) showing the association between rs9929218 in *CDH1* and the different non-syndromic orofacial cleft phenotypes; (A), all phenotypes combined; (B), cleft lip with or without cleft palate.


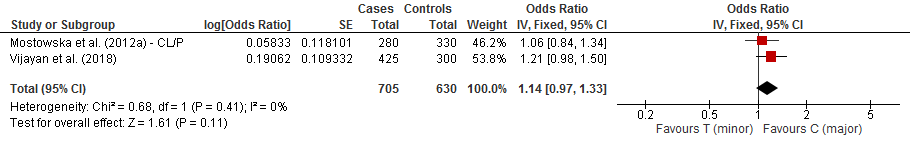


**Supplementary Figure S34.** Forest plot of the allelic genetic model analysis (minor allele vs. major allele) showing the association between rs35594616 in *DVL2* and all different non-syndromic orofacial cleft phenotypes combined.


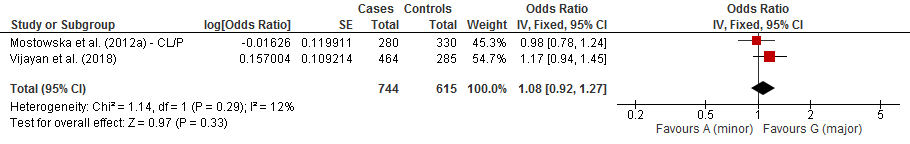


**Supplementary Figure S35.** Forest plot of the allelic genetic model analysis (minor allele vs. major allele) showing the association between rs2074222 in *DVL2* and all different non-syndromic orofacial cleft phenotypes combined.


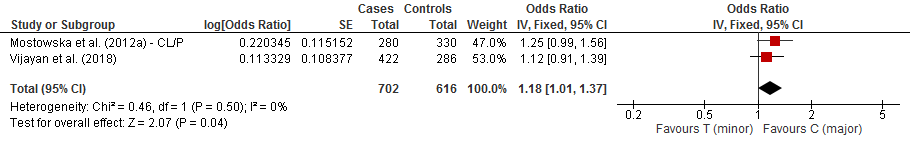


**Supplementary Figure S36.** Forest plot of the allelic genetic model analysis (minor allele vs. major allele) showing the association between rs222836 in *DVL2* and all different non-syndromic orofacial cleft phenotypes combined.


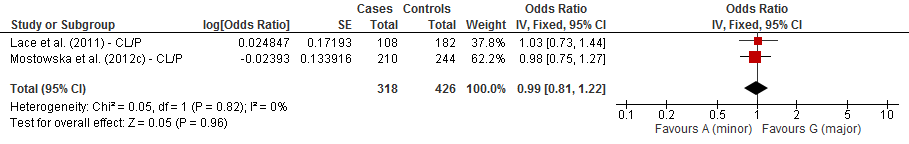


**Supplementary Figure S37.** Forest plot of the allelic genetic model analysis (minor allele vs. major allele) showing the association between rs12452064 in *WNT3* and non-syndromic cleft lip with or without cleft palate.

**A**


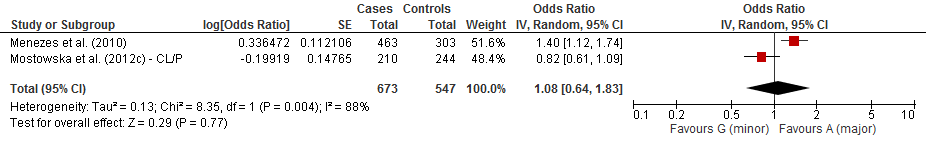


**B**


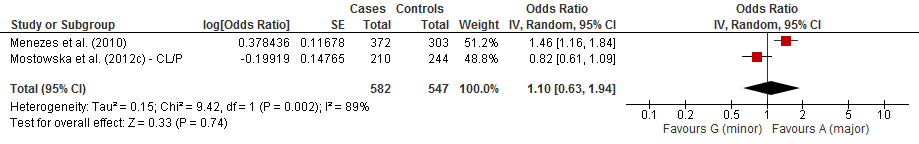


**Supplementary Figure S38.** Forest plots of the allelic genetic model analyses (minor allele vs. major allele) showing the association between rs9890413 near *WNT3* and the different non-syndromic orofacial cleft phenotypes; (A), all phenotypes combined; (B), cleft lip with or without cleft palate.

**A**


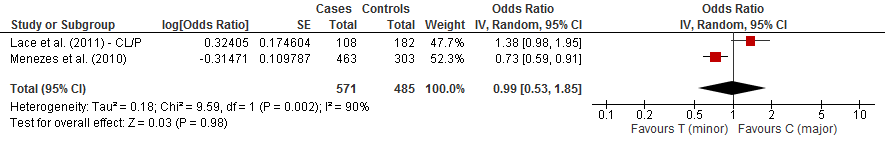


**B**


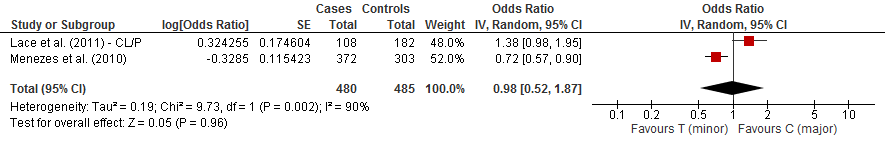


**Supplementary Figure S39.** Forest plots of the allelic genetic model analyses (minor allele vs. major allele) showing the association between rs111769 in *WNT3* and the different non-syndromic orofacial cleft phenotypes; (A), all phenotypes combined; (B), cleft lip with or without cleft palate.


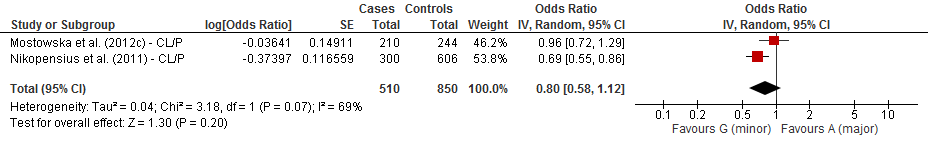


**Supplementary Figure S40.** Forest plot of the allelic genetic model analysis (minor allele vs. major allele) showing the association between rs4968282 in *WNT9B* and non-syndromic cleft lip with or without cleft palate.

**A**


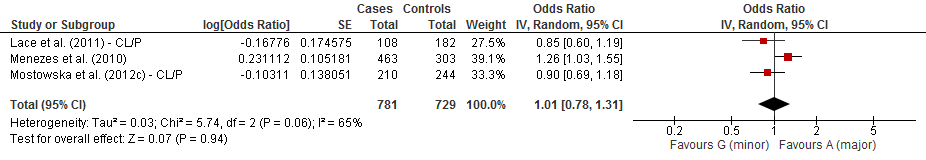


**B**


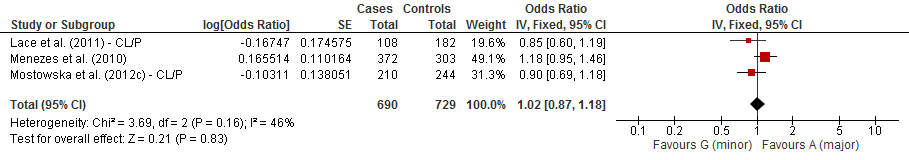


**Supplementary Figure S41.** Forest plots of the allelic genetic model analyses (minor allele vs. major allele) showing the association between rs2165846 in *WNT9B* and the different non-syndromic orofacial cleft phenotypes; (A), all phenotypes combined; (B), cleft lip with or without cleft palate.

**A**


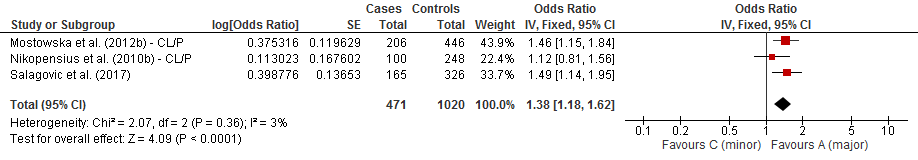


**B**


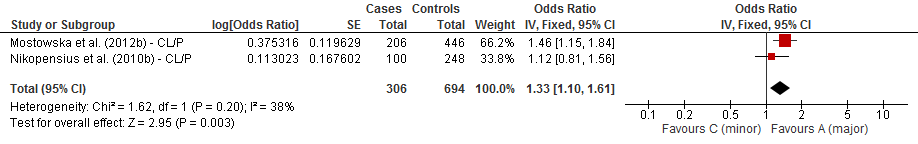


**Supplementary Figure S42.** Forest plots of the allelic genetic model analyses (minor allele vs. major allele) showing the association between rs227731 near *NOG* and the different non-syndromic orofacial cleft phenotypes; (A), all phenotypes combined; (B), cleft lip with or without cleft palate.

**A**


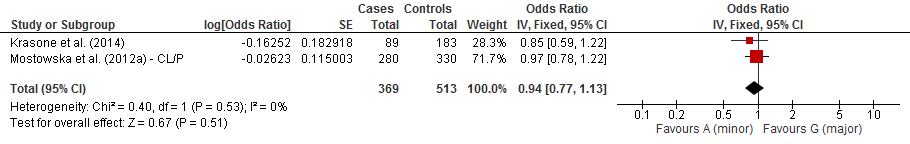


**B**


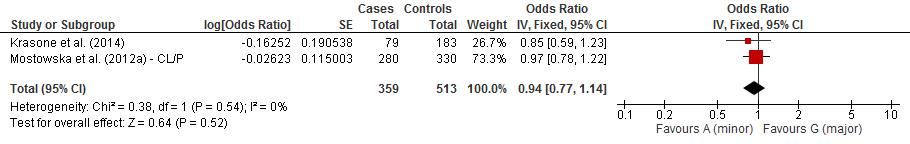


**Supplementary Figure S43.** Forest plots of the allelic genetic model analyses (minor allele vs. major allele) showing the association between rs2240308 in *AXIN2* and the different non-syndromic orofacial cleft phenotypes; (A), all phenotypes combined; (B), cleft lip with or without cleft palate.


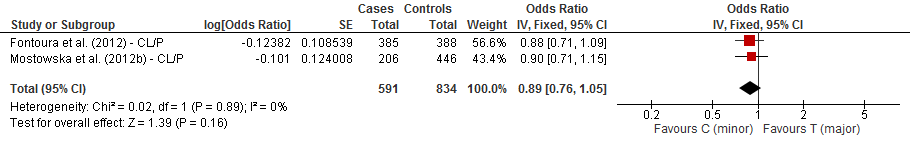


**Supplementary Figure S44.** Forest plot of the allelic genetic model analysis (minor allele vs. major allele) showing the association between rs13041247 near *MAFB* and non-syndromic cleft lip with or without cleft palate.


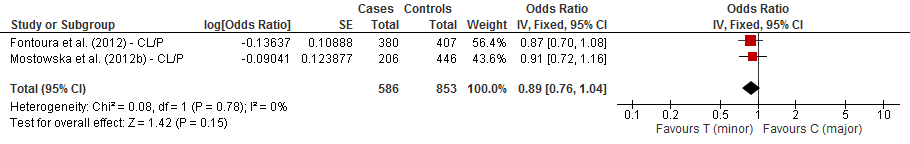


**Supplementary Figure S45.** Forest plot of the allelic genetic model analysis (minor allele vs. major allele) showing the association between rs11696257 near *MAFB* and non-syndromic cleft lip with or without cleft palate.

**A**


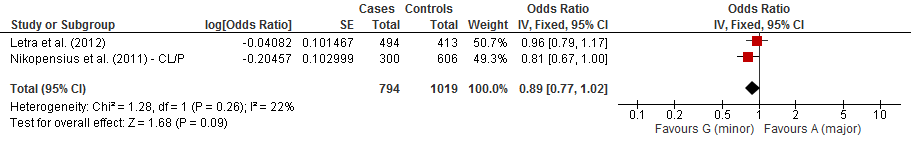


**B**


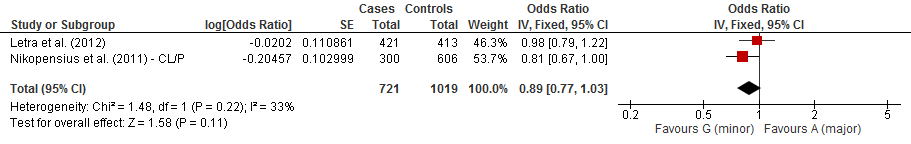


**Supplementary Figure S46.** Forest plots of the allelic genetic model analyses (minor allele vs. major allele) showing the association between rs17576 in *MMP9* and the different non-syndromic orofacial cleft phenotypes; (A), all phenotypes combined; (B), cleft lip with or without cleft palate.

**A**


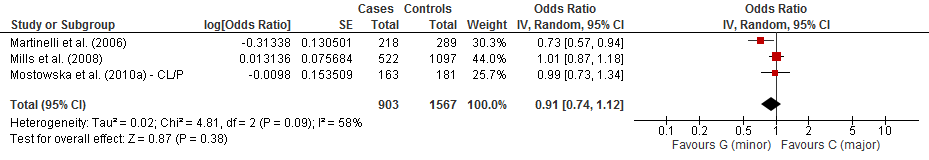


**B**


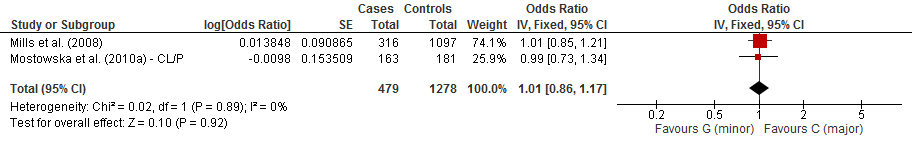


**Supplementary Figure S47.** Forest plots of the allelic genetic model analyses (minor allele vs. major allele) showing the association between rs1801198 in *TCN2* and the different non-syndromic orofacial cleft phenotypes; (A), all phenotypes combined; (B), cleft lip with or without cleft palate.
