# Supplementary material for: Genetic markers for non-syndromic orofacial clefts in populations of European ancestry: a meta-analysis
Source: Sci Rep. 2022 Jan 24;12:1214. doi: 10.1038/s41598-021-02159-5 (PMC8786890; doi:10.1038/s41598-021-02159-5)
Supplement: Supplementary file 4 — Supplementary Information 4. [file 41598_2021_2159_MOESM4_ESM.docx]

**Supplementary Information**

**Supplementary Tables S1, S2, S4, S5 and S6**

Supplementary Table S3 is provided in a separate file

**Genetic markers for non-syndromic orofacial clefts in populations of European ancestry: a meta-analysis**

Lara Slavec^1,2^, Nataša Karas Kuželički^2^, Igor Locatelli^3^, Ksenija Geršak^1,4*^

^1^ University Medical Centre Ljubljana, Division of Gynaecology and Obstetrics, Research Unit, Ljubljana, Slovenia

^2^ University of Ljubljana, Faculty of Pharmacy, Department of Clinical Biochemistry, Ljubljana, Slovenia

^3^ University of Ljubljana, Faculty of Pharmacy, Department of Social Pharmacy, Ljubljana, Slovenia

^4^ University of Ljubljana, Faculty of Medicine, Department of Gynaecology and Obstetrics, Ljubljana, Slovenia

***Corresponding author:**

Prof. Ksenija Geršak, MD, PhD

Tel: +386-1-522-6038

Email: ksenija.gersak@mf.uni-lj.si

**Supplementary Table S1** Characteristics of the 43 studies included in the meta-analysis

| Study | Population | Gene/loci name | Genotyping method | Cases and controls | NOS |
| --- | --- | --- | --- | --- | --- |
| Ardinger et al. (1989) | US American | *NR3C1* (*GRL*), *ESR1* (*ER*), *EGF*, *EGFR*, *TGFA* | PCR-RFLP | 80 (CL/P); 102 controls | 7 |
| Beaty et al. (1997) | US American | *TGFA* | PCR-RFLP | 130 (53 CLP, 26 CLO, 51 CPO); 87 controls | 7 |
| Birnbaum et al. (2009a) | Central European | *IRF6* | MALDI-TOF MS based | 460 (CL/P); 952 controls | 7 |
| Birnbaum et al. (2009b) | Central European | 8q24 | MALDI-TOF MS based | 462 (CL/P); 954 controls | 7 |
| Brandalize et al. (2007) | Brazilian | *MTHFR*, *MTR*, *MTRR* | PCR-RFLP | 114 (OFC); 100 controls | 9 |
| Carter et al. (2010) | Irish | *CLPTM1, CRISPLD2, FGFR2, GABRB3, GLI2, PTCH1, RARA, RYK, SATB2, SUMO1, TGFA, IRF6* | KASP assay | 472 (CL/P); 293 (CPO); 902 controls | 7 |
| Chevrier et al. (2007) | French | *MTHFR* | ASO-PCR, PCR-RFLP | 148 (CL/P); 59 (CPO); 168 controls | 9 |
| Chorna et al. (2011) | Ukrainian | *MTHFR*, *MTR,* *MTRR* | PCR-RFLP | 33 (OFC); 50 controls | 6 |
| Christensen et al. (1999) | Danish | *TGFA* | PCR-RFLP, PCR-SSCP | 192 (CL/P); 65 (CPO); 457 controls | 8 |
| Fontoura et al. (2012) | Brazilian | *MAFB, ABCA4* | Taqman assay | 400 (CL/P); 412 controls | 8 |
| Gaspar et al. (2004) | Brazilian | *MTHFR* | PCR-RFLP | 340 (OFC); 474 controls | 8 |
| Holder et al. (1992) | British | *TGFA* | PCR-RFLP | 60 (CL/P); 60 controls | 6 |
| Hozyasz et al. (2014a) | Polish | *CDH1* | Taqman assay | 250 (CL/P); 540 controls | 6 |
| Hwang et al. (1995) | US American | *TGFA* | PCR-RFLP | 183 (114 CL/P, 69 CPO); 284 controls | 6 |
| Jagomagi et al. (2010) | Estonian | *MTHFR, IRF6, SKI, MSX1, EDN1, TBX10, TGFB3, JAG2, BCL3, NECTIN2* | Microarray (APEX-2) | 100 (CL/P); 205 controls | 9 |
| Karas Kuželički et al. (2018) | Slovenian | *SLC19A1* (*RFC1*), *DHFR*, *FPGS*, *BHMT*, *GNMT*, *DNMT3B*, *MTHFD1*, *MTHFR*, *MTRR* | HRM analysis, Taqman assay | 172 (103 CL/P, 69 CPO); 199 controls | 9 |
| Krasone et al. (2014) | Latvian | *AXIN2, CDH1*, *IRF6* | Taqman assay | 93 (22 CLO, 61 CLP, 10 CPO); 190 controls | 7 |
| Lace et al. (2011) | Latvian | *SLC39A7*, *COL11A2*, *RING1*, *FGFR1*, *WNT3*, *WNT9B* | Microarray (APEX-2) | 108 (CL/P); 182 controls | 8 |
| Leslie et al. (2016b) | US American, Danish, Norwegian | *GRHL3*, *YAP1*, *PRKN* (*PARK2*) | TaqMan assay | 246 (CPO); 1685 controls | 6-9 |
| Letra et al. (2012) | Brazilian | *MMP2, MMP3, MMP7, MMP9, MMP10, MMP13, MMP14, MMP16, MMP25, MMP27, TIMP1, TIMP2* | TaqMan assay | 494 (10 CLO, 411 CLP, 73 CPO); 413 controls | 7 |
| Lidral et al. (1998) | US American | *TGFA*, *MSX1*, *TGFB3* | PCR-SSCP, sequencing | 243 (CL/P); 77 (CPO); 275 controls | 6 |
| Little et al. (2008) | English, Scottish | *MTHFR* | HRM analysis, MS-PCR | 190 (112 CL/P, 78 CPO); 248 controls | 9 |
| Ludwig et al. (2014) | Central European | *FOXE1* | MALDI-TOF MS based | 1104 (949 CL/P, 155 CPO); 1163 controls | 8 |
| Mangold et al. (2016) | Central European (p1) | *GRHL3* | KASP assay, TaqMan | (p1) 96 (CPO); 267 controls | 6 |
|  | Latvian (p2) |  | assay, direct sequencing | (p2) 51 (CPO); 94 controls | 7 |
|  | English (p3) |  |  | (p3) 94 (CPO); 177 controls | 6 |
| Martinelli et al. (2006) | Italian | *TCN2* | PCR-RFLP | 218 (OFC); 289 controls | 6 |
| Menezes et al. (2008) | Brazilian | *FGF3*, *FGF7*, *FGF10*, *FGF18*, *FGFR1*, *FGFR2* | TaqMan assay | 379 (326 CL/P, 53 CPO); 281 controls | 6 |
| Menezes et al. (2010) | Brazilian | *WNT3A*, *WNT5A*, *WNT8A*, *WNT11*, *WNT3*, *WNT9B* | Taqman assay | 463 (OFC); 372 (CL/P); 69 (CPO); 303 controls | 6 |
| Mills et al. (2008) | Irish | *MTHFD1*, *MTHFR*, *TCN2* | PCR-RFLP, ASO-PCR, MALDI-TOF MS based | 494 (CL/P), 321 (CPO); 1599 controls | 7 |
| Mitchell et al. (2001) | Danish | *TGFB3*, *MSX1* | Direct sequencing, PCR-SSCP | 198 (CL/P); 68 (CPO); 473 controls | 7 |
| Mostowska et al. (2010a) | Polish | *CBS, MTHFD1, MTHFR, MTR, MTRR, TCN2, BHMT, BHMT2, CHDH, CHKA, PCYT1A, PEMT* | PCR-RFLP, HRM analysis, PCR with electrophoresis | 174 (CL/P); 176 controls | 8 |
| Mostowska et al. (2010b) | Polish | *FOXE1*, *IRF6*, *MSX1*, *PAX9*, *TBX10*, *FGF10*, *FGFR1*, *TGFA*, *TGFB3*, *SUMO1*, 8q24 | PCR-RFLP | 175 (CL/P); 565 controls | 8 |
| Mostowska et al. (2012a) | Polish | *APC, AXIN1, AXIN2, CTNNB1, DVL2*, *GSK3B* | PCR-RFLP, HRM analysis | 280 (CL/P); 330 controls | 8 |
| Mostowska et al. (2012b) | Polish | *ABCA4*, *VAX1*, *NOG, MAFB* | HRM analysis | 206 (CL/P); 446 controls | 8 |
| Mostowska et al. (2012c) | Polish | *WNT3, WNT3A, WNT5A, WNT8A, WNT9B, WNT11* | PCR‐RFLP, HRM analysis | 210 (CL/P); 244 controls | 8 |
| Mostowska et al. (2015) | Polish | *GREM1* | PCR-RFLP | 334 (CL/P); 955 controls | 9 |
| Nikopensius et al. (2009) | Estonian (p1), Lithuanian (p2) | 8q24 | MALDI-TOF MS based | (p1) 163 (105 CL/P, 58 CPO); 1023 controls; (p2) 112 (CL/P); 244 controls | 6 |
| Nikopensius et al. (2010b) | Estonian | *THADA*, *VAX1*, *NOG*, 13q31.1, 15q13.3 | MALDI-TOF MS based | 101 (CL/P); 254 controls | 8 |
| Nikopensius et al. (2011) | Estonian, Latvian, Lithuanian | *IRF6*, *TGFA*, *FN1*, *MSX1*, *FGF2*, *FGF1*, *EDN1*, *FGFR1*, *FOXE1*, *MMP13*, *MMP25*, *WNT9B*, *TIMP2*, *NECTIN2* (*PVRL2)*, *BMP2*, *MMP9* | Microarray (APEX-2) | 300 (66 CLO, 234 CLP); 606 controls | 8 |
| Pezzetti et al. (2004) | Italian | *MTHFR* | PCR-RFLP | 110 (OFC); 289 controls | 6 |
| Salagovic et al. (2017) | Slovak | 8q24, *VAX1*, *NOG,* *IRF6* | HRM analysis | 165 (OFC); 326 controls | 7 |
| Shaw et al. (1998) | US American | *MTHFR* | PCR-RFLP | 310 (OFC); 383 controls | 8 |
| Van Rooij et al. (2003) | Dutch | *MTHFR* | PCR-RFLP | 130 (OFC); 150 controls. | 7 |
| Vijayan et al. (2018) | Brazilian | *AXIN1*, *APC*, *CTNNB1*, *DVL2*, *GSK3B* | Taqman assay | 471 (OFC); 504 controls | 7 |

Non-syndromic orofacial cleft: CL/P, cleft lip with or without cleft palate; CLO, cleft lip only; CPO, cleft palate only; CLP, cleft lip, alveolus and palate; OFC, all orofacial cleft phenotypes combined; NOS, study quality score by Newcastle-Ottawa scale. Abbreviations of genotyping methods and gene names are given in Supplementary Table S4.

**Supplementary Table S2** Characteristics of the 41 studies included in the systematic review but not in the meta-analysis

| First author (year) | Population | Gene/loci name | Genotyping method | Case and control samples | NOS | Association^a^ |
| --- | --- | --- | --- | --- | --- | --- |
| Al Chawa et al. (2014) ^1^ | Central European | *GREM1*, *NOG* | Sequencing | 192 (CL/P); 192 controls | 6 | N |
| Blanton et al. (2002)* ^2^ | US American, British, Czech | *MTHFR* | PCR-RFLP | 75 (OFC); 50 controls | 4 | N |
| Bohmer et al. (2018) ^3^ | Central European | 1q44, 2q21.1, 4q35.2, 5q14.1, 6p24.3, 6q15, 7q11.21, 10p11.21, 10q23.2, 10q23.31, 12p13.33, 14q24.3, 16q12.2, 17q24.1, 18p11.31, 18q12.2, 19p13.3 | MALDI-TOF MS based | 224 (CL/P); 986 controls | 7 | N |
| Boogaard et al. (2008) ^4^ | Dutch | *MSX1* | PCR, sequencing | 176 (OFC); 146 controls | 9 | N |
| Carta et al. (2012) ^5^ | British | *SUMO1, SUMO3, PIAS1, PIAS2* | Sequencing | 192 (CL/P); 192 controls | 7 | N |
| Chenevix-Trench et al. (1992)* ^6^ | Australian | *TGFA*, *RARA*, *BCL2*, *HOXB4* (*HOX2F*), *HOXB3* (*HOX2G*)*, EN2* | PCR-RFLP | 117 (CL/P); 113 controls | 5 | Y/N |
| Chevrier et al. (2005) ^7^ | French | *ADH1C* | ASO-PCR based | 205 (145 CL/P, 60 CPO); 115 controls | 7 | Y |
| Chevrier et al. (2008) ^8^ | French | *GSTM1, GSTT1* | ASO-PCR | 240 (164 CL/P, 76 CPO); 236 controls | 9 | N |
| de Assis et al. (2011) ^9^ | Central European | *SUMO1* | MALDI-TOF MS based, DHPLC | 413 (nsCL/P); 412 controls | 6 | N |
| Gaczkowska et al. (2018) ^10^ | Polish | *CDKAL1* | HRM analysis | 240 (CL/P); 445 controls | 7 | N |
| Hozyasz et al. (2010) ^11^ | Polish | *SLC25A13, ASS1,* *ASL* | PCR-RFLP, HRM analysis | 172 (CL/P); 188 controls | 7 | Y/N |
| Hozyasz et al. (2014b) ^12^ | Polish | *PAH*, *SLC7A5* (*LAT1*) | HRM analysis | 263 (CL/P); 270 controls | 9 | Y/N |
| Hozyasz et al. (2016) ^13^ | Polish | *GCH1* | TaqMan assay, HRM analysis, PCR-RFLP | 281 (CL/P); 574 controls | 7 | Y/N |
| Jugessur et al. (2008) ^14^ | Norwegian | *IRF6* | TaqMan assay | 573 (377 CL/P, 196 CPO); 763 controls | 7 | Y/N |
| Kempa et al. (2014) ^15^ | Latvian (p1), Lithuanian (p2) | *BMP4* | TaqMan assay | (p1) 164 (127 CL/P, 37 CPO); 190 controls; (p2) 119 (91 CL/P, 28 CPO); 99 controls | 7 | Y/N |
| Krapels et al. (2008) ^16^ | Dutch | *GSTP1* | PCR-RFLP | 94 (CL/P); 131 controls | 9 | N |
| Lace et al. (2012) ^17^ | Latvian | *BCL3* | MALDI-TOF MS based | 173 (132 CL/P, 41 CPO); 190 controls | 7 | N |
| Leslie et al. (2012) ^18^ | US American | *ARHGAP29* | Sequencing | 280 (CL/P); 456 controls | 7 | N |
| Leslie et al. (2016a) ^19^ | European | 2p24, 6p21, 10q26.3, 17q23, 19q13 | TaqMan assay | 607 (CL/P); 1685 controls | 6-9 | Y/N |
| Lie et al. (2008) ^20^ | Norwegian | *GSTT1, GSTM1* | ASO-PCR based | 573 (377 CL/P, 196 CPO); 763 controls | 7 | N |
| Ludwig et al. (2017) ^21^ | German | 1p31.1, 1q32.3, 1q44, 2p21, 2p16.2, 2q35, 5q14.1, 5q33.3, 6p25.1, 6p24.3, 6p22.3, 6p21.33, 6q25.2, 7q36.1, 8q21.13, 8q22.3, 9q22.2, 10q22.1, 11q23.3, 11q25, 12p11.21, 12q15, 12q23.3, 13q32.3, 14q12, 14q22.1, 14q24.3, 14q32.2, 17q24.1, 19p13.3 | MALDI-TOF MS based | 224 (CL/P); 921 controls | 6 | Y/N |
| Machado et al. (2019) ^22^ | Brazilian | *PON1*, *PON2*, *PON3* | TaqMan assay | 722 (515 CL/P, 207 CPO); 866 controls | 8 | Y/N |
| Machida et al. (2009) ^23^ | US American | *BSPRY, LINC01989, CCL2* | Sequencing | 90 (CL/P); 90 controls | 5 | N |
| Mansilla et al. (2006) ^24^ | US American | *PTCH1* | Sequencing | 90 (CL/P); 95 controls | 7 | Y/N |
| Minguzzi et al. (2012) ^25^ | Irish | *MTHFD1L* | Melting curve analysis | 878 (531 CL/P, 347 CPO); 1008 controls | 7 | N |
| Mostowska et al. (2014) ^26^ | Polish | *ATM, BLM, BRCA1, BRIP1, E2F1, MLH1, MRE11* (*MRE11A*)*, MSH2, MSH6, NBN, RAD50, RAD51* | TaqMan assays | 263 (CL/P); 526 controls | 9 | Y/N |
| Mostowska et al. (2018) ^27^ | Polish | *DLG1* | HRM analysis | 224 (175 CLP, 49 CLO); 473 controls | 7 | Y/N |
| Nasser et al. (2012) ^28^ | Central European | *VAX1* | Sequencing | 384 (CL/P); 384 controls | 6 | N |
| Nikopensius et al. (2010a) ^29^ | Estonian, Latvian, Lithuanian | *SKI*, *IRF6*, *TGFA*, *MSX1*, *FGF2*, *FGF1*, *COL11A2*, *RING1*, *FGFR1*, *COL2A1*, *JAG2*, *WNT3*, *TIMP2*, *LOXHD1* (*OFC11*), *NECTIN2* (*PVRL2*), *CLPTM1*, *BMP2*, *TIMP3* | Microarray (APEX-2) | 104 (CPO); 606 controls | 8 | Y/N |
| Rahimov et al. (2008)* ^30^ | Norwegian, Danish | *IRF6* | TaqMan assay | 513 (368 CL/P, 145 CPO); 1245 controls | 6 | Y |
| Romitti et al. (1999) ^31^ | US American | *TGFA*, *TGFB3*, *MSX1* | PCR-RFLP, PCR with electrophoresis | 214 (154 CL/P, 60 CPO); 373 controls | 8 | N |
| Sassani et al. (1993)* ^32^ | US American | *TGFA* | PCR-RFLP, HRM analysis | 83 (CL/P); 84 controls | 5 | Y/N |
| Scapoli et al. (2006) ^33^ | Italian | *NECTIN1* (*PVRL1*) | PCR-SSCP, sequencing | 143 (CL/P); 292 controls | 6 | Y/N |
| Shiang et al. (1993)* ^34^ | US American | *TGFA* | PCR-RFLP, PCR-SSCP, DGGE | 52 (CPO); 192 controls | 6 | Y/N |
| Sozen et al. (2009a)* ^35^ | US American, Australian | *NECTIN1* (*PVRL1*) | Sequencing | 216 (OFC); 223 controls | 6 | Y/N |
| Sozen et al. (2009b) ^36^ | US American | *PVR, NECTIN2 (PVRL2)* | PCR-SSCP, sequencing | 73 (CL/P); 105 controls | 5 | N |
| Stoll et al. (1993)* ^37^ | French | *TGFA* | PCR-RFLP | 98 (CL/P); 57 (CPO); 99 controls | 9 | Y/N |
| Stoll et al. (2004) ^38^ | German | *TGFB1* | Melting curve analysis | 60 (CL/P); 60 controls | 9 | Y/N |
| Turhani et al. (2005)* ^39^ | Austrian | *CLPTM1, NECTIN1 (PVRL1)* | Sequencing | 25 (CL/P); 25 controls | 7 | Y/N |
| Van Rooij et al. (2001) ^40^ | Dutch | *GSTT1*, *CYP1A1* | PCR with electrophoresis, PCR-RFLP | 113 (OFC); 104 controls | 8 | N |
| Vintiner et al. (1993) ^41^ | British | *RARA*, *VIM* | PCR-RFLP | 61 (CL/P); 60 controls | 6 | N |

Non-syndromic orofacial clefts: CL/P, cleft lip with or without cleft palate; CLO, cleft lip only; CPO, cleft palate only; CLP, cleft lip, alveolus and palate; OFC, all orofacial cleft phenotypes combined; NOS, study quality score by Newcastle-Ottawa scale

^a^, Y, positive association for all the studied genetic markers; N, negative association for all the studied genetic markers; Y/N, positive association for at least one of the genetic markers studied;

*, studies that were additionally excluded from the meta-analysis (studies with NOS ≤5, studies with controls that deviated from HWE, or studies where not enough data were available for HWE calculation);

Abbreviations of the genotyping methods and the gene names are given in Supplementary Table S4.

**References of studies included in the systematic review but not in the meta-analysis**

1. Al Chawa, T. *et al.* Nonsyndromic cleft lip with or without cleft palate: increased burden of rare variants within Gremlin-1, a component of the bone morphogenetic protein 4 pathway. *Birth Defects Res. Part A - Clin. Mol. Teratol.* **100**, 493–498; https://doi.org/10.1002/bdra.23244 (2014).

2. Blanton, S. H., Patel, S., Hecht, J. T. & Mulliken, J. B. MTHFR is not a risk factor in the development of isolated nonsyndromic cleft lip and palate. *Am. J. Med. Genet.* **110**, 404–405; https://doi.org/10.1002/ajmg.10496 (2002).

3. Böhmer, A. C. *et al.* Investigation of dominant and recessive inheritance models in genome-wide association studies data of nonsyndromic cleft lip with or without cleft palate. *Birth Defects Res.* **110**, 336–341; https://doi.org/10.1002/bdr2.1144 (2018).

4. Boogaard, M. J. H. *et al.* The MSX1 allele 4 homozygous child exposed to smoking at periconception is most sensitive in developing nonsyndromic orofacial clefts. *Hum. Genet.* **124**, 525–534; https://doi.org/10.1007/s00439-008-0569-6 (2008).

5. Carta, E. *et al.* Investigation of SUMO pathway genes in the etiology of nonsyndromic cleft lip with or without cleft palate. *Birth Defects Res. Part A - Clin. Mol. Teratol.* **94**, 459–463; https://doi.org/10.1002/bdra.23008 (2012).

6. Chenevix-Trench, G., Jones, K., Green, A. C., Duffy, D. L. & Martin, N. G. Cleft lip with or without cleft palate: Associations with transforming growth factor alpha and retinoic acid receptor loci. *Am. J. Hum. Genet.* **51**, 1377–1385; (1992).

7. Chevrier, C. *et al.* Interaction between the ADH1C polymorphism and maternal alcohol intake in the risk of nonsyndromic oral clefts: an evaluation of the contribution of child and maternal genotypes. *Birth Defects Res. Part A - Clin. Mol. Teratol.* **73**, 114–122; https://doi.org/10.1002/bdra.20103 (2005).

8. Chevrier, C. *et al.* Genetic susceptibilities in the association between maternal exposure to tobacco smoke and the risk of nonsyndromic oral cleft. *Am. J. Med. Genet. Part A* **146**, 2396–2406; https://doi.org/10.1002/ajmg.a.32505 (2008).

9. de Assis, N. A. *et al.* SUMO1 as a candidate gene for non-syndromic cleft lip with or without cleft palate: no evidence for the involvement of common or rare variants in central European patients. *Int. J. Pediatr. Otorhinolaryngol.* **75**, 49–52; https://doi.org/10.1016/j.ijporl.2010.10.005 (2011).

10. Gaczkowska, A. *et al.* Association of CDKAL1 nucleotide variants with the risk of non-syndromic cleft lip with or without cleft palate. *J. Hum. Genet.* **63**, 397–406; https://doi.org/10.1038/s10038-017-0397-4 (2018).

11. Hozyasz, K. K., Mostowska, A., Wojcicki, P., Lianeri, M. & Jagodzinski, P. P. Polymorphic variants of genes related to arginine metabolism and the risk of orofacial clefts. *Arch. Oral Biol.* **55**, 861–866; https://doi.org/10.1016/j.archoralbio.2010.07.012 (2010).

12. Hozyasz, K. K. *et al.* Association of common variants in PAH and LAT1 with non-syndromic cleft lip with or without cleft palate (NSCL/P) in the Polish population. *Arch. Oral Biol.* **59**, 363–369; https://doi.org/10.1016/j.archoralbio.2014.01.003 (2014b).

13. Hozyasz, K. K. *et al.* Nucleotide variants of the BH4 biosynthesis pathway gene GCH1 and the risk of orofacial clefts. *Mol. Neurobiol.* **53**, 769–776; https://doi.org/10.1007/s12035-015-9342-8 (2016).

14. Jugessur, A. *et al.* Genetic variants in IRF6 and the risk of facial clefts: single-marker and haplotype-based analyses in a population-based case-control study of facial clefts in Norway. *Genet. Epidemiol.* **32**, 413–424; https://doi.org/10.1002/gepi.20314 (2008).

15. Kempa, I. *et al.* Association of BMP4 polymorphisms with non-syndromic cleft lip with or without cleft palate and isolated cleft palate in Latvian and Lithuanian populations. *Stomatologija* **16**, 94–101; (2014).

16. Krapels, I. P. C. *et al.* The I105V polymorphism in glutathione S-transferase P1, parental smoking and the risk for nonsyndromic cleft lip with or without cleft palate. *Eur. J. Hum. Genet.* **16**, 358–366; https://doi.org/10.1038/sj.ejhg.5201973 (2008).

17. Lace, B. *et al.* BCL3 gene role in facial morphology. *Birth Defects Res. Part A - Clin. Mol. Teratol.* **94**, 918–924; https://doi.org/10.1002/bdra.23085 (2012).

18. Leslie, E. J. *et al.* Expression and mutation analyses implicate ARHGAP29 as the etiologic gene for the cleft lip with or without cleft palate locus identified by genome-wide association on chromosome 1p22. *Birth Defects Res. Part A - Clin. Mol. Teratol.* **94**, 934–942; https://doi.org/10.1002/bdra.23076 (2012).

19. Leslie, E. J. *et al.* A multi-ethnic genome-wide association study identifies novel loci for non-syndromic cleft lip with or without cleft palate on 2p 24.2, 17q23 and 19q13. *Hum. Mol. Genet.* **25**, 2862–2872; https://doi.org/10.1093/hmg/ddw104 (2016a).

20. Lie, R. T. *et al.* Maternal smoking and oral clefts the role of detoxification pathway genes. *Epidemiology* **19**, 606–615; https://doi.org/10.1097/EDE.0b013e3181690731 (2008).

21. Ludwig, K. U. *et al.* Imputation of orofacial clefting data identifies novel risk loci and sheds light on the genetic background of cleft lip ± cleft palate and cleft palate only. *Hum. Mol. Genet.* **26**, 829–842; https://doi.org/10.1093/hmg/ddx012 (2017).

22. Machado, R. A. *et al.* Interactions between superoxide dismutase and paraoxonase polymorphic variants in nonsyndromic cleft lip with or without cleft palate in the Brazilian population. *Environ. Mol. Mutagen.* **60**, 185–196; https://doi.org/10.1002/em.22239 (2019).

23. Machida, J. *et al.* Searching for genes for cleft lip and/or palate based on breakpoint analysis of a balanced translocation t(9;17)(q32;q12). *Cleft Palate-Craniofacial J.* **46**, 532–540; https://doi.org/10.1597/08-047.1 (2009).

24. Mansilla, M. A. *et al.* Contributions of PTCH gene variants to isolated cleft lip and palate. *Cleft Palate. Craniofac. J.* **43**, 21–29; (2006).

25. Minguzzi, S. *et al.* Genotyping of a tri-allelic polymorphism by a novel melting curve assay in MTHFD1L: an association study of nonsyndromic cleft in Ireland. *BMC Med. Genet.* **13**, 1–8; https://doi.org/10.1186/1471-2350-13-29 (2012).

26. Mostowska, A. *et al.* Genetic variants in BRIP1 (BACH1) contribute to risk of nonsyndromic cleft lip with or without cleft palate. *Birth Defects Res. Part A - Clin. Mol. Teratol.* **100**, 670–678; https://doi.org/10.1002/bdra.23275 (2014).

27. Mostowska, A. *et al.* Common variants in DLG1 locus are associated with non-syndromic cleft lip with or without cleft palate. *Clin. Genet.* **93**, 784–793; https://doi.org/10.1111/cge.13141 (2018).

28. Nasser, E. *et al.* Resequencing of VAX1 in patients with nonsyndromic cleft lip with or without cleft palate. *Birth Defects Res. Part A - Clin. Mol. Teratol.* **94**, 925–933; https://doi.org/10.1002/bdra.23078 (2012).

29. Nikopensius, T. *et al.* Genetic variants in COL2A1, COL11A2, and IRF6 contribute risk to nonsyndromic cleft palate. *Birth Defects Res. Part A - Clin. Mol. Teratol.* **88**, 748–756; https://doi.org/10.1002/bdra.20700 (2010a).

30. Rahimov, F. *et al.* Disruption of an AP-2α binding site in an IRF6 enhancer is strongly associated with cleft lip. *Nat. Genet.* **40**, 1341–1347; https://doi.org/10.1038/ng.242.Disruption (2008).

31. Romitti, P. *et al.* Candidate genes for nonsyndromic cleft lip and palate and maternal cigarette smoking and alcohol consumption: evaluation of genotype-environment interactions from a population-based case-control study of orofacial clefts. *Teratology* **59**, 39–50; https://doi.org/10.1002/(SICI)1096-9926(199901)59:1<39::AID-TERA9>3.0.CO;2-7 (1999).

32. Sassani, R. *et al.* Association between alleles of the transforming growth factor-alpha locus and the occurrence of cleft lip. *Am. J. Med. Genet.* **45**, 565–569; https://doi.org/10.1002/ajmg.1320450508 (1993).

33. Scapoli, L. *et al.* Study of the PVRL1 gene in Italian nonsyndromic cleft lip patients with or without cleft palate. *Ann. Hum. Genet.* **70**, 410–413; https://doi.org/10.1111/j.1529-8817.2005.00237.x (2006).

34. Shiang, R. *et al.* Association of transforming growth-factor α gene polymorphisms with nonsyndromic cleft palate only (CPO). *Am. J. Hum. Genet.* **53**, 836–843; (1993).

35. Sözen, M. A., Hecht, J. T. & Spritz, R. A. Mutation analysis of the PVRL1 gene in caucasians with nonsyndromic cleft lip/palate. *Genet. Test. Mol. Biomarkers* **13**, 617–621; https://doi.org/10.1089/gtmb.2009.0052 (2009a).

36. Sözen, M. A., Hecht, J. T. & Spritz, R. A. Mutation and association analysis of the PVR and PVRL2 genes in patients with non-syndromic cleft lip and palate. *Genet. Mol. Biol.* **32**, 466–469; https://doi.org/10.1590/S1415-47572009000300007 (2009b).

37. Stoll, C., Qian, J. F., Feingold, J., Sauvage, P. & May, E. Genetic variation in transforming growth factor α: possible association of BamHI polymorphism with bilateral sporadic cleft lip and palate. *Hum. Genet.* **92**, 81–82; https://doi.org/10.1007/BF00216150 (1993).

38. Stoll, C. *et al.* Analysis of polymorphic TGFB1 codons 10, 25, and 263 in a German patient group with non-syndromic cleft lip, alveolus, and palate compared with healthy adults. *BMC Med. Genet.* **9**, 1–9; (2004).

39. Turhani, D. *et al.* Mutation analysis of CLPTM 1 and PVRL 1 genes in patients with non-syndromic clefts of lip, alveolus and palate. *J. Cranio-Maxillofacial Surg.* **33**, 301–306; https://doi.org/10.1016/j.jcms.2005.04.004 (2005).

40. Van Rooij, I. A. L. M. *et al.* Smoking, genetic polymorphisms in biotransformation enzymes, and nonsyndromic oral clefting: a gene-environment interaction. *Epidemiology* **12**, 502–507; (2001).

41. Vintiner, G. M., Lo, K. K., Holder, S. E., Winter, R. M. & Malcolm, S. Exclusion of candidate genes from a role in cleft lip with or without cleft palate: linkage and association studies. *J. Med. Genet.* **30**, 773–778; https://doi.org/10.1136/jmg.30.9.773 (1993).

**Supplementary Table S4** Abbreviations of genotyping methods (A) and gene names (B)

**A**

| **Method abbreviation** | **Description** |
| --- | --- |
| APEX-2 | Arrayed primer extension reaction |
| ASO-PCR | Allele-specific oligonucleotide–polymerase chain reaction |
| DGGE | Denaturing gradient gel electrophoresis |
| DHPLC | Denaturing high pressure liquid chromatography |
| HRM | High resolution melting |
| KASP | Kompetitive allele specific polymerase chain reaction |
| MALDI-TOF MS | Matrix assisted laser desorption ionization–time of flight mass spectrometry |
| MS-PCR | Mutagenically separated–polymerase chain reaction |
| PCR | Polymerase chain reaction |
| PCR-RFLP | Polymerase chain reaction–restriction fragment length polymorphism |
| PCR-SSCP | Polymerase chain reaction–single-strand conformation polymorphism |

**B**

| **Gene symbol** | **Gene name** |
| --- | --- |
| *ABCA4* | ATP binding cassette subfamily A member 4 |
| *ADH1C* | Alcohol dehydrogenase 1C (class I), gamma polypeptide |
| *APC* | APC regulator of WNT signaling pathway |
| *ARHGAP29* | Rho GTPase activating protein 29 |
| *ASL* | Argininosuccinate lyase |
| *ASS1* | Argininosuccinate synthase 1 |
| *ATM* | ATM serine/threonine kinase |
| *AXIN1/2* | Axin 1/2 |
| *BCL2/3* | BCL2 (apoptosis regulator); BCL3 (transcription coactivator) |
| *BHMT/2* | Betaine--homocysteine S-methyltransferase/2 |
| *BLM* | BLM RecQ like helicase |
| *BMP2/4* | Bone morphogenetic protein 2/4 |
| *BRCA1* | BRCA1 DNA repair associated |
| *BRIP1* | BRCA1 interacting protein C-terminal helicase 1 |
| *BSPRY* | B-box and SPRY domain containing |
| *CBS* | Cystathionine-beta-synthase |
| *CCL2* | C-C motif chemokine ligand 2 |
| *CDH1* | Cadherin 1 |
| *CDKAL1* | CDK5 regulatory subunit associated protein 1 like 1 |
| *CHDH* | Choline dehydrogenase |
| *CHKA* | Choline kinase α |
| *CLPTM1* | CLPTM1 regulator of GABA type A receptor forward trafficking |
| *COL2A1/11A2* | Collagen type II alpha 1 chain/ type XI alpha 2 chain |
| *CRISPLD2* | Cysteine rich secretory protein LCCL domain containing 2 |
| *CTNNB1* | Catenin beta 1 |
| *CYP1A1* | Cytochrome P450 family 1 subfamily A member 1 |
| *DHFR* | Dihydrofolate reductase |
| *DLG1* | Discs large MAGUK scaffold protein 1 |
| *DNMT3B* | DNA methyltransferase 3 beta |
| *DVL2* | Dishevelled segment polarity protein 2 |
| *E2F1* | E2F transcription factor 1 |
| *EDN1* | Endothelin 1 |
| *EGF* | Epidermal growth factor |
| *EGFR* | Epidermal growth factor receptor |
| *EN2* | Engrailed homeobox 2 |
| *ESR1* (*ER*) | Estrogen receptor 1 |
| *FGF1-3/7/10/18* | Fibroblast growth factor 1-3/7/10/18 |
| *FGFR1/2* | Fibroblast growth factor receptor 1/2 |
| *FN1* | Fibronectin 1 |
| *FOXE1* | Forkhead box E1 |
| *FPGS* | Folylpolyglutamate synthase |
| *GABRB3* | γ-Aminobutyric acid type A receptor subunit beta3 |
| *GCH1* | GTP cyclohydrolase 1 |
| *GLI2* | GLI family zinc finger 2 |
| *GNMT* | Glycine N-methyltransferase |
| *GREM1* | Gremlin 1, DAN family BMP antagonist |
| *GRHL3* | Grainyhead like transcription factor 3 |
| *GSK3B* | Glycogen synthase kinase 3 beta |
| *GSTM1* | Glutathione S-transferase mu 1 |
| *GSTP1* | Glutathione S-transferase pi 1 |
| *GSTT1* | Glutathione S-transferase theta 1 |
| *HOXB3/4* (*HOX2G/F*) | Homeobox B3/B4 |
| *IRF6* | Interferon regulatory factor 6 |
| *JAG2* | Jagged canonical Notch ligand 2 |
| *LINC01989* | Long intergenic non-protein coding RNA 1989 |
| *LOXHD1* (*OFC11*) | Lipoxygenase homology domains 1 |
| *MAFB* | MAF bZIP transcription factor B |
| *MLH1* | MutL homolog 1 |
| *MMP2/3/7/9/10/13/14/16/25/27* | Matrix metallopeptidase 2/3/7/9/10/13/14/16/25/27 |
| *MRE11* (*MRE11A*) | MRE11 homolog, double strand break repair nuclease |
| *MSH2/6* | MutS homolog 2/6 |
| *MSX1* | Msh homeobox 1 |
| *MTHFD1* | Methylenetetrahydrofolate dehydrogenase, cyclohydrolase and formyltetrahydrofolate synthetase 1 |
| *MTHFD1L* | Methylenetetrahydrofolate dehydrogenase (NADP+ dependent) 1 like |
| *MTHFR* | Methylenetetrahydrofolate reductase |
| *MTR* | 5-Methyltetrahydrofolate-homocysteine methyltransferase |
| *MTRR* | 5-Methyltetrahydrofolate-homocysteine methyltransferase reductase |
| *NBN* | Nibrin |
| *NECTIN1/2* (*PVRL1/2*) | Nectin cell adhesion molecule 1/2 |
| *NOG* | Noggin |
| *NR3C1* (*GRL*) | Nuclear receptor subfamily 3 group C member 1 |
| *PAH* | Phenylalanine hydroxylase |
| *PAX9* | Paired box 9 |
| *PCYT1A* | Phosphate cytidylyltransferase 1, choline, alpha |
| *PEMT* | Phosphatidylethanolamine N-methyltransferase |
| *PIAS1/2* | Protein inhibitor of activated STAT 1/2 |
| *PON1-3* | Paraoxonase 1-3 |
| *PRKN* (*PARK2*) | Parkin RBR E3 ubiquitin protein ligase |
| *PTCH1* | Patched 1 |
| *PVR* | PVR cell adhesion molecule |
| *RAD50* | RAD50 double strand break repair protein |
| *RAD51* | RAD51 recombinase |
| *RARA* | Retinoic acid receptor α |
| *RING1* | Ring finger protein 1 |
| *RYK* | Receptor like tyrosine kinase |
| *SATB2* | SATB homeobox 2 |
| *SKI* | SKI proto-oncogene |
| *SLC7A5* (*LAT1*) | Solute carrier family 7 member 5 |
| *SLC19A1* (*RFC1*) | Solute carrier family 19 member 1 |
| *SLC25A13* | Solute carrier family 25 member 13 |
| *SLC39A7* | Solute carrier family 39 member 7 |
| *SUMO1/3* | Small ubiquitin like modifier 1/3 |
| *TBX10* | T-box transcription factor 10 |
| *TCN2* | Transcobalamin 2 |
| *TGFA/B1/B3* | Transforming growth factor α/β1/β3 |
| *THADA* | THADA armadillo repeat containing |
| *TIMP1-3* | TIMP metallopeptidase inhibitor 1-3 |
| *VAX1* | Ventral anterior homeobox 1 |
| *VIM* | Vimentin |
| *WNT3/3A/5A/8A/9B/11* | Wnt family member 3/3A/5A/8A/9B/11 |
| *YAP1* | Yes1 associated transcriptional regulator |

**Supplementary Table S5**  Results of the meta-analysis performed to determine the associations between the genetic markers and the different non-syndromic orofacial cleft phenotypes in populations of European ancestry; statistically non-significant findings

| **Gene** | **Variant^a^** | **A/a** | **Phenotype** | **N** | **N_CA_** | **N_CO_** | **Allelic model^b^** | | **Dominant model^c^** | | **Recessive model^d^** | | **Overdominant model^e^** | |
| --- | --- | --- | --- | --- | --- | --- | --- | --- | --- | --- | --- | --- | --- | --- |
|  |  |  |  |  |  |  | **OR (95% CI)** | **p-value** | **OR (95% CI)** | **p-value** | **OR (95% CI)** | **p-value** | **OR (95% CI)** | **p-value** |
| MTHFR | rs1801131 | A/C | OFC | 6 | 1181 | 1908 | 0.87 (0.72 - 1.06) | 0.167 | 0.97 (0.83 - 1.13) | 0.707 | 0.89 (0.68 - 1.16) | 0.383 | 1.01 (0.87 - 1.19) | 0.855 |
|  |  |  | CL/P | 3 | 610 | 1454 | 0.84 (0.63 - 1.12) | 0.238 | 0.98 (0.80 - 1.20) | 0.848 | 0.91 (0.63 - 1.31) | 0.602 | 1.01 (0.82 - 1.25) | 0.914 |
|  |  |  | CPO | 2 | 334 | 1249 | 1.04 (0.87 - 1.26) | 0.647 | 1.10 (0.87 - 1.40) | 0.431 | 0.94 (0.61 - 1.44) | 0.769 | 1.13 (0.89 - 1.44) | 0.318 |
|  | rs1801133 | C/T | OFC | 11 | 2413 | 3627 | 0.99 (0.92 - 1.07) | 0.858 | 0.95 (0.86 - 1.06) | 0.358 | 1.10 (0.93 - 1.30) | 0.283 | 0.92 (0.83 - 1.02) | 0.117 |
|  |  |  | CL/P | 5 | 1002 | 2361 | 0.97 (0.86 - 1.08) | 0.575 | 0.94 (0.81 - 1.09) | 0.410 | 1.02 (0.80 - 1.29) | 0.897 | 0.93 (0.80 - 1.09) | 0.376 |
|  |  |  | CPO | 4 | 518 | 2190 | 0.92 (0.80 - 1.07) | 0.276 | 0.91 (0.75 - 1.11) | 0.349 | 0.89 (0.65 - 1.22) | 0.473 | 0.97 (0.80 - 1.17) | 0.721 |
| MTR | rs1805087 | A/G | OFC | 3 | 307 | 319 | 1.11 (0.85 - 1.45) | 0.432 | 1.21 (0.87 - 1.67) | 0.255 | 0.88 (0.43 - 1.79) | 0.716 | 1.26 (0.90 - 1.76) | 0.173 |
| CTNNB1 | rs4533622 | C/A | OFC | 2 | 721 | 586 | 0.99 (0.85 - 1.16) | 0.936 | 1.01 (0.80 - 1.28) | 0.932 | 0.97 (0.74 - 1.29) | 0.850 | 1.03 (0.82 - 1.28) | 0.818 |
| MTRR | rs1801394 | A/G | OFC | 3 | 369 | 415 | 0.87 (0.61 - 1.23) | 0.421 | 1.05 (0.71 - 1.56) | 0.800 | 0.66 (0.32 - 1.38) | 0.269 | 1.62 (0.75 - 3.51) | 0.218 |
|  |  |  | CL/P | 2 | 267 | 365 | 0.99 (0.79 - 1.25) | 0.964 | 1.11 (0.73 - 1.70) | 0.619 | 0.92 (0.66 - 1.30) | 0.644 | 0.96 (0.60 - 1.53) | 0.852 |
| FGF10 | rs1448037 | G/A | OFC | 2 | 541 | 449 | 1.12 (0.82 - 1.54) | 0.486 | NA | NA | NA | NA | NA | NA |
|  |  |  | CL/P | 2 | 488 | 449 | 1.11 (0.78 - 1.57) | 0.560 | NA | NA | NA | NA | NA | NA |
| BHMT | rs3733890 | G/A | OFC | 2 | 345 | 374 | 0.90 (0.48 - 1.66) | 0.726 | 1.00 (0.53 - 1.88) | 0.995 | 0.42 (0.06 - 3.06) | 0.395 | 1.19 (0.88 - 1.60) | 0.253 |
|  |  |  | CL/P | 2 | 276 | 374 | 0.89 (0.48 - 1.67) | 0.723 | 1.00 (0.52 - 1.90) | 0.992 | 0.42 (0.06 - 3.06) | 0.392 | 1.17 (0.85 - 1.61) | 0.329 |
| APC | rs351771 | T/C | OFC | 2 | 728 | 702 | 1.09 (0.94 - 1.27) | 0.256 | 1.08 (0.88 - 1.34) | 0.459 | 1.21 (0.90 - 1.62) | 0.217 | 0.98 (0.80 - 1.21) | 0.869 |
| WNT8a | rs2040862 | C/T | OFC | 2 | 673 | 547 | 0.95 (0.75 - 1.20) | 0.686 | NA | NA | NA | NA | NA | NA |
|  |  |  | CL/P | 2 | 582 | 547 | 0.95 (0.75 - 1.21) | 0.704 | NA | NA | NA | NA | NA | NA |
| FGFR1 | rs328300 | T/G | CL/P | 2 | 282 | 342 | 0.99 (0.79 - 1.24) | 0.950 | NA | NA | NA | NA | NA | NA |
|  | rs6987534 | G/C | CL/P | 2 | 280 | 344 | 1.02 (0.81 - 1.28) | 0.886 | NA | NA | NA | NA | NA | NA |
| MTHFD1 | rs2236225 | C/T | OFC | 2 | 342 | 362 | 0.93 (0.65 - 1.31) | 0.662 | 0.84 (0.60 - 1.18) | 0.322 | 0.96 (0.50 - 1.86) | 0.907 | 0.88 (0.66 - 1.19) | 0.414 |
|  |  |  | CL/P | 2 | 273 | 362 | 0.85 (0.67 - 1.06) | 0.150 | 0.77 (0.54 - 1.10) | 0.155 | 0.81 (0.53 - 1.23) | 0.319 | 0.92 (0.67 - 1.27) | 0.611 |
| TGFB3 | rs3917200 | T/C | OFC | 2 | 351 | 486 | 1.02 (0.69 - 1.50) | 0.924 | NA | NA | NA | NA | NA | NA |
|  |  |  | CL/P | 2 | 257 | 486 | 0.98 (0.64 - 1.50) | 0.917 | NA | NA | NA | NA | NA | NA |
|  |  |  | CPO | 2 | 94 | 486 | 1.12 (0.61 - 2.05) | 0.723 | NA | NA | NA | NA | NA | NA |
|  | rs2205181 | T/C | CL/P | 2 | 268 | 365 | 1.04 (0.56 - 1.95) | 0.898 | NA | NA | NA | NA | NA | NA |
| CDH1 | rs9929218 | G/A | OFC | 2 | 343 | 724 | 1.02 (0.84 - 1.25) | 0.834 | 1.04 (0.80 - 1.35) | 0.760 | 0.99 (0.63 - 1.56) | 0.961 | 1.05 (0.81 - 1.36) | 0.734 |
|  |  |  | CL/P | 2 | 333 | 724 | 1.01 (0.82 - 1.23) | 0.957 | 1.01 (0.78 - 1.32) | 0.912 | 0.98 (0.62 - 1.56) | 0.944 | 1.02 (0.78 - 1.33) | 0.877 |
| WNT3 | rs12452064 | G/A | CL/P | 2 | 318 | 426 | 0.99 (0.81 - 1.22) | 0.958 | NA | NA | NA | NA | NA | NA |
|  | rs9890413 | A/G | OFC | 2 | 673 | 547 | 1.08 (0.64 - 1.83) | 0.773 | NA | NA | NA | NA | NA | NA |
|  |  |  | CL/P | 2 | 582 | 547 | 1.10 (0.63 - 1.94) | 0.738 | NA | NA | NA | NA | NA | NA |
|  | rs111769 | C/T | OFC | 2 | 571 | 485 | 0.99 (0.53 - 1.85) | 0.976 | NA | NA | NA | NA | NA | NA |
|  |  |  | CL/P | 2 | 480 | 485 | 0.98 (0.52 - 1.87) | 0.963 | NA | NA | NA | NA | NA | NA |
| WNT9B | rs4968282 | A/G | CL/P | 2 | 510 | 850 | 0.80 (0.58 - 1.12) | 0.195 | NA | NA | NA | NA | NA | NA |
|  | rs2165846 | A/G | OFC | 3 | 781 | 729 | 1.01 (0.78 - 1.31) | 0.941 | NA | NA | NA | NA | NA | NA |
|  |  |  | CL/P | 3 | 690 | 729 | 1.02 (0.87 - 1.18) | 0.833 | NA | NA | NA | NA | NA | NA |
| MAFB | rs13041247 | T/C | CL/P | 2 | 591 | 834 | 0.89 (0.76 - 1.05) | 0.163 | 0.82 (0.66 - 1.02) | 0.082 | 0.96 (0.68 - 1.35) | 0.804 | 0.84 (0.68 - 1.04) | 0.118 |
|  | rs11696257 | C/T | CL/P | 2 | 586 | 853 | 0.89 (0.76 - 1.04) | 0.155 | 0.83 (0.67 - 1.03) | 0.091 | 0.94 (0.67 - 1.32) | 0.717 | 0.85 (0.69 - 1.06) | 0.148 |
| MMP9 | rs17576 | A/G | OFC | 2 | 794 | 1019 | 0.89 (0.77 - 1.02) | 0.093 | NA | NA | NA | NA | NA | NA |
|  |  |  | CL/P | 2 | 721 | 1019 | 0.89 (0.77 – 1.03) | 0.114 | NA | NA | NA | NA | NA | NA |
| TCN2 | rs1801198 | C/G | OFC | 3 | 903 | 1567 | 0.91 (0.74 - 1.12) | 0.382 | 0.91 (0.77 - 1.09) | 0.323 | 0.89 (0.61 - 1.28) | 0.519 | 0.96 (0.82 - 1.14) | 0.669 |
|  |  |  | CL/P | 2 | 479 | 1278 | 1.01 (0.86 - 1.17) | 0.921 | 0.99 (0.78 - 1.25) | 0.931 | 1.04 (0.79 - 1.36) | 0.783 | 0.97 (0.78 - 1.20) | 0.767 |

A/a, major and minor alleles, where major allele is listed first; Phenotype, non-syndromic orofacial cleft phenotype studied (CL/P, cleft lip with or without cleft palate; CPO, cleft palate only; OFC, all phenotypes combined); N, number of studies included in the analysis (allelic model); N_CA_, total number of cases included in the analysis (allelic model); N_CO_, total number of controls included in the analysis (allelic model); NA, not available; OR (95% CI), pooled odds ratio with 95% confidence interval for each analysis

^a^, Genetic variants found in named genes or in their close proximity; listed based on chromosomal location; ^b^, minor allele (a) vs. major allele (A); ^c^, Aa+aa vs. AA; ^d^, aa vs. Aa+AA; ^e^, Aa vs. AA+aa. Abbreviations of gene names are given in Supplementary Table S4 and further details about the meta-analysis are presented in Supplementary Table S6**Supplementary Table S6** Evaluation of between-study heterogeneity and further details about the meta-analysis performed to determine the associations between 47 genetic markers and the different non-syndromic orofacial cleft phenotypes in populations of European ancestry

| **Gene** | **Variant^a^** | **Genomic location^b^** | **Phenotype** | **Allelic model^c^** | | | | | **Dominant model^d^** | | | | | **Recessive model^e^** | | | | | **Overdominant model^f^** | | | |
| --- | --- | --- | --- | --- | --- | --- | --- | --- | --- | --- | --- | --- | --- | --- | --- | --- | --- | --- | --- | --- | --- | --- |
|  |  | **(GRCh38.p13)** |  | **N** | **p (Q)** | **I² [%]** | **Model** | **N** | | **p (Q)** | **I² [%]** | **Model** | **N** | | **p (Q)** | **I² [%]** | **Model** | **N** | | **p (Q)** | **I² [%]** | **Model** |
| MTHFR | rs1801131 | chr1:11794419 | OFC | 6 | 0.072 | 50.7 | RE | 5 | | 0.496 | 0 | FE | 5 | | 0.542 | 0 | FE | 5 | | 0.869 | 0 | FE |
|  |  |  | CL/P | 3 | 0.063 | 63.9 | RE | 2 | | 0.811 | 0 | FE | 2 | | 0.895 | 0 | FE | 2 | | 0.891 | 0 | FE |
|  |  |  | CPO | 2 | 0.454 | 0 | FE | 2 | | 0.713 | 0 | FE | 2 | | 0.278 | 15.1 | FE | 2 | | 0.776 | 0 | FE |
|  | rs1801133 | chr1:11796321 | OFC | 11 | 0.154 | 30.7 | FE | 11 | | 0.200 | 25.6 | FE | 11 | | 0.384 | 6.3 | FE | 11 | | 0.612 | 0 | FE |
|  |  |  | CL/P | 5 | 0.286 | 20.2 | FE | 5 | | 0.272 | 22.4 | FE | 5 | | 0.572 | 0 | FE | 5 | | 0.564 | 0 | FE |
|  |  |  | CPO | 4 | 0.324 | 13.6 | FE | 4 | | 0.707 | 0 | FE | 4 | | 0.210 | 33.7 | FE | 4 | | 0.926 | 0 | FE |
| GRHL3 | rs41268753 | chr1:24342967 | CPO | 4 | 0.437 | 0 | FE | 3 | | 0.284 | 20.5 | FE | 0 | | NE | NE | NE | 3 | | 0.302 | 16.5 | FE |
| ABCA4 | rs560426 | chr1:94087882 | CL/P | 2 | 0.001 | 90.9 | RE | 2 | | 0.024 | 80.5 | RE | 2 | | 0.002 | 89.4 | RE | 2 | | 0.484 | 0 | FE |
|  | rs481931 | chr1:94104460 | CL/P | 2 | 0.711 | 0 | FE | 2 | | 0.257 | 22.1 | FE | 2 | | 0.428 | 0 | FE | 2 | | 0.049 | 74.2 | RE |
| IRF6 | rs642961 | chr1:209815925 | OFC | 4 | 0.758 | 0 | FE | 4 | | 0.984 | 0 | FE | 4 | | 0.166 | 41.0 | FE | 4 | | 0.956 | 0 | FE |
|  |  |  | CL/P | 3 | 0.497 | 0 | FE | 3 | | 0.872 | 0 | FE | 3 | | 0.117 | 53.4 | RE | 3 | | 0.983 | 0 | FE |
|  | rs2013162 | chr1:209795339 | OFC | 2 | 0.343 | 0 | FE | 2 | | 0.770 | 0 | FE | 2 | | 0.106 | 61.8 | RE | 2 | | 0.538 | 0 | FE |
|  |  |  | CL/P | 2 | 0.953 | 0 | FE | 2 | | 0.737 | 0 | FE | 2 | | 0.485 | 0 | FE | 2 | | 0.491 | 0 | FE |
|  | rs2235371 | chr1:209790735 | OFC | 2 | 0.838 | 0 | FE | 2 | | 0.998 | 0 | FE | 0 | | NE | NE | NE | 2 | | 0.824 | 0 | FE |
|  |  |  | CL/P | 2 | 0.752 | 0 | FE | 2 | | 0.757 | 0 | FE | 0 | | NE | NE | NE | 2 | | 0.757 | 0 | FE |
|  | rs590223 | chr1:209773362 | CL/P | 2 | 0.121 | 58.5 | RE | 0 | | NA | NA | NA | 0 | | NA | NA | NA | 0 | | NA | NA | NA |
| WNT3a | rs708111 | chr1:228003664 | OFC | 2 | 0.766 | 0 | FE | 0 | | NA | NA | NA | 0 | | NA | NA | NA | 0 | | NA | NA | NA |
|  |  |  | CL/P | 2 | 0.996 | 0 | FE | 0 | | NA | NA | NA | 0 | | NA | NA | NA | 0 | | NA | NA | NA |
|  | rs752107 | chr1:228059650 | OFC | 2 | 0.880 | 0 | FE | 0 | | NA | NA | NA | 0 | | NA | NA | NA | 0 | | NA | NA | NA |
|  |  |  | CL/P | 2 | 0.649 | 0 | FE | 0 | | NA | NA | NA | 0 | | NA | NA | NA | 0 | | NA | NA | NA |
| MTR | rs1805087 | chr1:236885200 | OFC | 3 | 0.869 | 0 | FE | 3 | | 0.924 | 0 | FE | 3 | | 0.582 | 0 | FE | 3 | | 0.749 | 0 | FE |
| TGFA | TaqI | - | OFC | 5 | 0.005 | 73.5 | RE | 4 | | 0.016 | 71.0 | RE | 2 | | 0.178 | 44.9 | FE | 4 | | 0.165 | 41.1 | FE |
|  |  |  | CL/P | 5 | 0.010 | 70.1 | RE | 4 | | 0.025 | 67.9 | RE | 2 | | 0.089 | 65.4 | RE | 4 | | 0.200 | 35.4 | FE |
|  |  |  | CPO | 4 | 0.317 | 15.0 | FE | 3 | | 0.203 | 37.3 | FE | 0 | | NE | NE | NE | 3 | | 0.226 | 32.8 | FE |
|  | BamHI | - | CL/P | 2 | 0.075 | 68.4 | RE | 2 | | 0.159 | 49.5 | FE | 0 | | NE | NE | NE | 2 | | 0.346 | 0 | FE |
|  | RsaI | - | CL/P | 2 | 0.725 | 0 | FE | 2 | | 0.726 | 0 | FE | 2 | | 0.656 | 0 | FE | 2 | | 0.916 | 0 | FE |
| CTNNB1 | rs4533622 | chr3:41200847 | OFC | 2 | 0.834 | 0 | FE | 2 | | 0.703 | 0 | FE | 2 | | 0.976 | 0 | FE | 2 | | 0.702 | 0 | FE |
| WNT5a | rs566926 | chr3:55486750 | OFC | 2 | 0.557 | 0 | FE | 0 | | NA | NA | NA | 0 | | NA | NA | NA | 0 | | NA | NA | NA |
|  |  |  | CL/P | 2 | 0.397 | 0 | FE | 0 | | NA | NA | NA | 0 | | NA | NA | NA | 0 | | NA | NA | NA |
| MTRR | rs1801394 | chr5:7870860 | OFC | 3 | 0.087 | 59.0 | RE | 2 | | 0.797 | 0 | FE | 3 | | 0.007 | 79.7 | RE | 3 | | 0.003 | 83.2 | RE |
|  |  |  | CL/P | 2 | 0.461 | 0 | FE | 2 | | 0.962 | 0 | FE | 2 | | 0.270 | 17.7 | FE | 2 | | 0.788 | 0 | FE |
| FGF10 | rs1448037 | chr5:44352242 | OFC | 2 | 0.055 | 72.9 | RE | 0 | | NA | NA | NA | 0 | | NA | NA | NA | 0 | | NA | NA | NA |
|  |  |  | CL/P | 2 | 0.087 | 65.9 | RE | 0 | | NA | NA | NA | 0 | | NA | NA | NA | 0 | | NA | NA | NA |
| BHMT | rs3733890 | chr5:79126136 | OFC | 2 | 0.007 | 86.3 | RE | 2 | | 0.032 | 78.2 | RE | 2 | | 0.006 | 86.8 | RE | 2 | | 0.406 | 0 | FE |
|  |  |  | CL/P | 2 | 0.011 | 84.5 | RE | 2 | | 0.044 | 75.3 | RE | 2 | | 0.008 | 85.7 | RE | 2 | | 0.422 | 0 | FE |
| APC | rs351771 | chr5:112828864 | OFC | 2 | 0.324 | 0 | FE | 2 | | 0.454 | 0 | FE | 2 | | 0.328 | 0 | FE | 2 | | 0.873 | 0 | FE |
| WNT8a | rs2040862 | chr5:138084300 | OFC | 2 | 0.533 | 0 | FE | 0 | | NA | NA | NA | 0 | | NA | NA | NA | 0 | | NA | NA | NA |
|  |  |  | CL/P | 2 | 0.515 | 0 | FE | 0 | | NA | NA | NA | 0 | | NA | NA | NA | 0 | | NA | NA | NA |
| FGFR1 | rs328300 | chr8:38486981 | CL/P | 2 | 0.513 | 0 | FE | 0 | | NA | NA | NA | 0 | | NA | NA | NA | 0 | | NA | NA | NA |
|  | rs6987534 | chr8:38442197 | CL/P | 2 | 0.415 | 0 | FE | 0 | | NA | NA | NA | 0 | | NA | NA | NA | 0 | | NA | NA | NA |
| 8q24 | rs987525 | chr8:128933908 | OFC | 5 | 0.039 | 60.5 | RE | 5 | | 0.008 | 70.8 | RE | 5 | | 0.468 | 0 | FE | 5 | | 0.066 | 54.6 | RE |
|  |  |  | CL/P | 4 | 0.331 | 12.4 | FE | 4 | | 0.131 | 46.7 | FE | 4 | | 0.508 | 0 | FE | 4 | | 0.351 | 8.4 | FE |
| FOXE1 | rs4460498 | chr9:97858130 | OFC | 2 | 0.258 | 21.8 | FE | 2 | | 0.125 | 57.5 | RE | 2 | | 1.000 | 0 | FE | 2 | | 0.174 | 45.8 | FE |
|  |  |  | CL/P | 2 | 0.184 | 43.3 | FE | 2 | | 0.099 | 63.3 | RE | 2 | | 0.831 | 0 | FE | 2 | | 0.184 | 43.4 | FE |
|  | rs3758249 | chr9:97851858 | OFC | 2 | 0.168 | 47.3 | FE | 2 | | 0.032 | 78.3 | RE | 2 | | 0.576 | 0 | FE | 2 | | 0.025 | 80.1 | RE |
|  |  |  | CL/P | 2 | 0.118 | 59.0 | RE | 2 | | 0.025 | 80.2 | RE | 2 | | 0.716 | 0 | FE | 2 | | 0.028 | 79.3 | RE |
| VAX1 | rs7078160 | chr10:117068049 | OFC | 3 | 0.454 | 0 | FE | 3 | | 0.691 | 0 | FE | 3 | | 0.282 | 20.9 | FE | 3 | | 0.855 | 0 | FE |
|  |  |  | CL/P | 2 | 0.399 | 0 | FE | 2 | | 0.461 | 0 | FE | 2 | | 0.876 | 0 | FE | 2 | | 0.642 | 0 | FE |
| MTHFD1 | rs2236225 | chr14:64442127 | OFC | 2 | 0.097 | 63.8 | RE | 2 | | 0.237 | 28.4 | FE | 2 | | 0.087 | 66.0 | RE | 2 | | 0.757 | 0 | FE |
|  |  |  | CL/P | 2 | 0.372 | 0 | FE | 2 | | 0.460 | 0 | FE | 2 | | 0.381 | 0 | FE | 2 | | 0.951 | 0 | FE |
| TGFB3 | rs3917200 | chr14:75963525 | OFC | 2 | 0.619 | 0 | FE | 0 | | NA | NA | NA | 0 | | NA | NA | NA | 0 | | NA | NA | NA |
|  |  |  | CL/P | 2 | 0.632 | 0 | FE | 0 | | NA | NA | NA | 0 | | NA | NA | NA | 0 | | NA | NA | NA |
|  |  |  | CPO | 2 | 0.731 | 0 | FE | 0 | | NA | NA | NA | 0 | | NA | NA | NA | 0 | | NA | NA | NA |
|  | rs2205181 | chr14:75987776 | CL/P | 2 | 0.008 | 85.7 | RE | 0 | | NA | NA | NA | 0 | | NA | NA | NA | 0 | | NA | NA | NA |
| GREM1 | rs1258763 | chr15:32758222 | CL/P | 2 | 0.688 | 0 | FE | 2 | | 0.417 | 0 | FE | 2 | | 0.746 | 0 | FE | 2 | | 0.375 | 0 | FE |
| CDH1 | rs9929218 | chr16:68787043 | OFC | 2 | 0.588 | 0 | FE | 2 | | 0.477 | 0 | FE | 2 | | 0.999 | 0 | FE | 2 | | 0.468 | 0 | FE |
|  |  |  | CL/P | 2 | 0.769 | 0 | FE | 2 | | 0.688 | 0 | FE | 2 | | 0.967 | 0 | FE | 2 | | 0.665 | 0 | FE |
| DVL2 | rs35594616 | chr17:7226521 | OFC | 2 | 0.411 | 0 | FE | 2 | | 0.818 | 0 | FE | 2 | | 0.033 | 78.1 | RE | 2 | | 0.112 | 60.4 | RE |
|  | rs2074222 | chr17:7226655 | OFC | 2 | 0.285 | 12.4 | FE | 2 | | 0.772 | 0 | FE | 2 | | 0.005 | 87.1 | RE | 2 | | 0.037 | 77.1 | RE |
|  | rs222836 | chr17:7229843 | OFC | 2 | 0.499 | 0 | FE | 2 | | 0.019 | 81.9 | RE | 2 | | 0.300 | 6.7 | FE | 2 | | 0.005 | 87.5 | RE |
| WNT3 | rs12452064 | chr17:46790821 | CL/P | 2 | 0.823 | 0 | FE | 0 | | NA | NA | NA | 0 | | NA | NA | NA | 0 | | NA | NA | NA |
|  | rs9890413 | chr17:46824083 | OFC | 2 | 0.004 | 88.0 | RE | 0 | | NA | NA | NA | 0 | | NA | NA | NA | 0 | | NA | NA | NA |
|  |  |  | CL/P | 2 | 0.002 | 89.4 | RE | 0 | | NA | NA | NA | 0 | | NA | NA | NA | 0 | | NA | NA | NA |
|  | rs111769 | chr17:46794621 | OFC | 2 | 0.002 | 89.6 | RE | 0 | | NA | NA | NA | 0 | | NA | NA | NA | 0 | | NA | NA | NA |
|  |  |  | CL/P | 2 | 0.002 | 89.7 | RE | 0 | | NA | NA | NA | 0 | | NA | NA | NA | 0 | | NA | NA | NA |
| WNT9B | rs4968282 | chr17:46881571 | CL/P | 2 | 0.074 | 68.6 | RE | 0 | | NA | NA | NA | 0 | | NA | NA | NA | 0 | | NA | NA | NA |
|  | rs2165846 | chr17:46864000 | OFC | 3 | 0.057 | 65.2 | RE | 0 | | NA | NA | NA | 0 | | NA | NA | NA | 0 | | NA | NA | NA |
|  |  |  | CL/P | 3 | 0.158 | 45.8 | FE | 0 | | NA | NA | NA | 0 | | NA | NA | NA | 0 | | NA | NA | NA |
| NOG | rs227731 | chr17:56695877 | OFC | 3 | 0.355 | 3.3 | FE | 3 | | 0.833 | 0 | FE | 3 | | 0.152 | 46.9 | FE | 3 | | 0.475 | 0 | FE |
|  |  |  | CL/P | 2 | 0.203 | 38.4 | FE | 2 | | 0.602 | 0 | FE | 2 | | 0.135 | 55.3 | RE | 2 | | 0.492 | 0 | FE |
| AXIN2 | rs2240308 | chr17:65558473 | OFC | 2 | 0.528 | 0 | FE | 2 | | 0.735 | 0 | FE | 2 | | 0.416 | 0 | FE | 2 | | 0.664 | 0 | FE |
|  |  |  | CL/P | 2 | 0.540 | 0 | FE | 2 | | 0.660 | 0 | FE | 2 | | 0.485 | 0 | FE | 2 | | 0.833 | 0 | FE |
| MAFB | rs13041247 | chr20:40640434 | CL/P | 2 | 0.890 | 0 | FE | 2 | | 0.903 | 0 | FE | 2 | | 0.709 | 0 | FE | 2 | | 0.760 | 0 | FE |
|  | rs11696257 | chr20:40642176 | CL/P | 2 | 0.780 | 0 | FE | 2 | | 0.857 | 0 | FE | 2 | | 0.444 | 0 | FE | 2 | | 0.557 | 0 | FE |
| MMP9 | rs17576 | chr20:46011586 | OFC | 2 | 0.257 | 22.0 | FE | 0 | | NA | NA | NA | 0 | | NA | NA | NA | 0 | | NA | NA | NA |
|  |  |  | CL/P | 2 | 0.223 | 32.6 | FE | 0 | | NA | NA | NA | 0 | | NA | NA | NA | 0 | | NA | NA | NA |
| TCN2 | rs1801198 | chr22:30615623 | OFC | 3 | 0.090 | 58.4 | RE | 3 | | 0.241 | 29.7 | FE | 3 | | 0.116 | 53.5 | RE | 3 | | 0.894 | 0 | FE |
|  |  |  | CL/P | 2 | 0.895 | 0 | FE | 2 | | 0.720 | 0 | FE | 2 | | 0.850 | 0 | FE | 2 | | 0.648 | 0 | FE |

Phenotype, non-syndromic orofacial cleft phenotype studied (CL/P, cleft lip with or without cleft palate; CPO, cleft palate only; OFC, all phenotypes combined); N, number of studies included in the analysis; p (Q), p-value of Cochrane Q test for evaluation of between-study heterogeneity; I², I² statistic for evaluation of between-study heterogeneity; Model, model used to pool the odds ratios (FE, fixed-effects model; RE, random-effects model); NA, not available; NE, not estimable;

^a^, Genetic variants found in named genes or in their close proximity; ^b^, exact genomic location based on dbSNP database; ^c^, minor allele (a) vs. major allele (A); ^d^, Aa+aa vs. AA; ^e^, aa vs. Aa+AA; ^f^, Aa vs. AA+aa;

Abbreviations of gene names are given in Supplementary Material S4
